# Specialized activities and expression differences for *Clostridium thermocellum* biofilm and planktonic cells

**DOI:** 10.1038/srep43583

**Published:** 2017-02-27

**Authors:** Alexandru Dumitrache, Dawn M. Klingeman, Jace Natzke, Miguel Rodriguez Jr, Richard J. Giannone, Robert L. Hettich, Brian H. Davison, Steven D. Brown

**Affiliations:** 1 BioEnergy Science Center (BESC), Oak Ridge National Laboratory, Oak Ridge, TN, 37831, U.S.A.; 2 Biosciences Division, Oak Ridge National Laboratory, Oak Ridge, TN, 37831, U.S.A.; 3 Chemical Sciences Division, Oak Ridge National Laboratory, Oak Ridge, TN, 37831, U.S.A.

## Abstract

*Clostridium (Ruminiclostridium) thermocellum* is a model organism for its ability to deconstruct plant biomass and convert the cellulose into ethanol. The bacterium forms biofilms adherent to lignocellulosic feedstocks in a continuous cell-monolayer in order to efficiently break down and uptake cellulose hydrolysates. We developed a novel bioreactor design to generate separate sessile and planktonic cell populations for omics studies. Sessile cells had significantly greater expression of genes involved in catabolism of carbohydrates by glycolysis and pyruvate fermentation, ATP generation by proton gradient, the anabolism of proteins and lipids and cellular functions critical for cell division consistent with substrate replete conditions. Planktonic cells had notably higher gene expression for flagellar motility and chemotaxis, cellulosomal cellulases and anchoring scaffoldins, and a range of stress induced homeostasis mechanisms such as oxidative stress protection by antioxidants and flavoprotein co-factors, methionine repair, Fe-S cluster assembly and repair in redox proteins, cell growth control through tRNA thiolation, recovery of damaged DNA by nucleotide excision repair and removal of terminal proteins by proteases. This study demonstrates that microbial attachment to cellulose substrate produces widespread gene expression changes for critical functions of this organism and provides physiological insights for two cells populations relevant for engineering of industrially-ready phenotypes.

*Clostridium (Ruminiclostridium) thermocellum* is a fermentative anaerobic thermophile that is being engineered for use in the consolidated bioprocessing of second-generation bio-feedstocks into liquid fuels and other chemicals^1^,^2^. Though the gram-positive bacterium is a versatile cellulose and hemicellulose hydrolyzer, it converts only the resulting cellodextrins to ethanol and organic acids. When co-cultured with a five-carbon sugar fermenting microbe, mutant *C. thermocellum* strains approached industrially relevant ethanol yields and titers of up to 80% and 38 g/L respective^3^ making it a good candidate for further engineering.

*C. thermocellum* secretes self-assembled, carbohydrate-active, mobile^4^ or cell-bound cellulosomes^5^ that mediate attachment to solid cellulosic substrates through carbohydrate binding domains. Once attached, sugar solubilization through cellulosome-directed carbohydrate hydrolysis is synergized with the active uptake of resulting oligomeric cellodextrans that serve as the organism’ primary energy source^6^. Thus, at the aqueous-cellulose interface, the bacterium produces unique and atypical biofilms of a single layer of cells which were able to capture 71% to 86% of cellulose hydrolysates although it lacks the encapsulating glycoproteic matrix of canonical biofilms^7^. Through division the sessile cells (SS) of the biofilm release free, non-adherent planktonic cells (PL) in the aqueous environment at rates that closely matched, in non-limited conditions, the rate of cellulose consumption^8^. Implicitly, planktonic cells can recolonize free cellulose; however, as substrates deplete and biofilms erode the balance shifts to increasingly large planktonic populations. For example, in a mixed cellulolytic consortium, the planktonic fraction represented three quarters of the total population^9^. The features of more conventional biofilms that do not use the substratum as the carbon source have been reviewed recently^10^.

Although co-existing free and attached bacteria contribute to a balanced culture, in various species they were found to express different gene sets in response to individual microenvironment challenges and the diffusion and flux of nutrients^11^,^12^,^13^,^14^,^15^. In *C. thermocellum*, biofilms specialized for growth on crystalline cellulose and capable of nutrient capture thus create conditions mimicking continuous carbon limitation for planktonic cells; a phenomenon best exemplified by the low detection of soluble glucans during fermentation^16^. Continued strain development for eventual industrial application requires a robust understanding of *C. thermocellum*’s cellular physiology. Although gene expression in *C. thermocellum* was described for growth on cellulose^17^, switchgrass and poplar^18^,^19^ and after induced chemical stress^20^,^21^ the discrete analysis of the two cell populations has not yet been performed. Here we set out to compare differential gene expression by whole transcriptomic and proteomic analyses of sessile and planktonic samples isolated simultaneously at peak culture activity and assess their adaptation to substrate availability and the contributions they bring to bioconversion and culture growth.

## Results and Discussion

**Figure 1:**
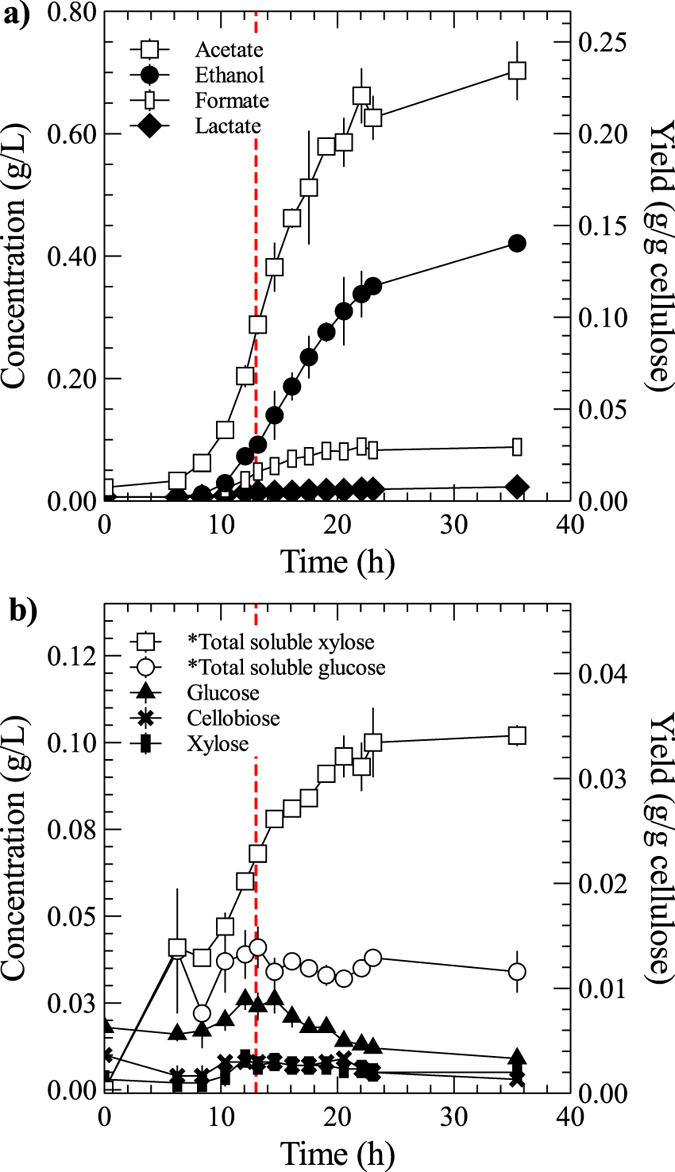
Concentration and yield (normalized to initial mass of cellulose) of fermentation products (**a**) and soluble sugars (*total glucose and xylose include the soluble oligomeric forms) in the culture supernatant (**b**). Cellulose rapidly converted to products (in ~12 h), while planktonic cells had very limited access to soluble sugars. Starting cellulose (filter paper) concentration of 3 g/L. Vertical red line marks the corresponding time for RNAseq and proteomic sample collection in subsequent replicate fermentations. Averaged data of biological duplicate fermentations.

Cultures of *Clostridium (Ruminiclostridium) thermocellum* were grown in batch bioreactors with controlled mixing, pH, temperature and nitrogen gas purge with solid Whatman paper #3 as the cellulose carbon source. Therefore, sessile (SS) and planktonic (PL) cells fractions were exposed to equal growth conditions with differences only in the availability of solid attachment support and access to products of cellulose hydrolysis. For biofilm isolation from the aqueous planktonic fraction samples were collected by rapid removal of the cellulose solids with adherent cells in a mesh pocket from a novel reactor design, described below. To determine the optimal point for omics sample collection and population comparison, a preliminary time-course analysis of culture growth to end-point cellulose conversion was performed. Initial cellulose at 3 g/L was hydrolyzed and fermented to near completion within 24 hours after inoculation with an estimated 7 h culture lag phase. Acetate and ethanol were produced at typical wild-type yields with a mass to mass ratio of 2:1.2 and low levels of formate were measured (Fig. 1), consistent with fast and otherwise normal fermentative wild-type culture growth (e.g., ethanol at 25% of theoretical yield). To verify whether planktonic cell fractions were limited by carbohydrate availability, a time-course analysis of the concentration of soluble monomeric and oligomeric cellulose hydrolysates was performed. Total soluble hexose sugars (in glucose equivalents) were well below 1.5% of the initial cellulose concentration (Fig. 1) confirming that hydrolysates which bypass capture by the biofilm were rapidly consumed by resident planktonic fractions. Therefore, the planktonic cells exist in a continuous carbon-limited state. These represented the primary conditions that were hypothesized to generate cell populations with different genetic expression.

**Figure 2:**
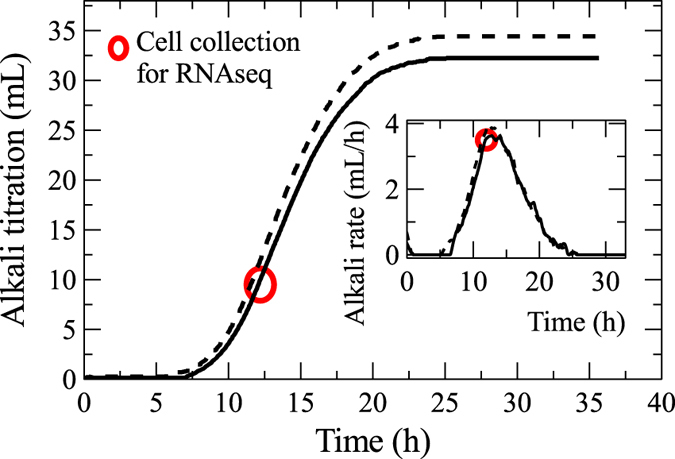
Alkali titration (mL) and rate of titration (mL/h) of biological replicate fermentations (solid and dash-line, respectively). Cell fraction collection for RNA-Seq analysis (red circle) was made at peak titration rate, analogous to a high rate of culture fermentative activity.

From a bioreactor, co-existent sessile and planktonic cell fractions were collected (in four independent biological replicate fermentations) for RNA-Seq analysis ~7 hours after exiting the lag phase and shortly before reaching the highest point of culture activity (Fig. 2) at peak alkali titration rate – a common, reliable metric of fermentative product formation^22^. This ensured that biofilms did not reach the growth limitations imposed by maximally colonized cellulose surfaces, as defined in previous studies^8^. At the point of RNA collection, the planktonic and sessile cell abundances were measured at 1:4 ratio, with an average of 0.37 trillion to 1.44 trillion cells per population per reactor assuming similar population protein contents, respectively (Fig. S1). Beyond that point, as bioconversion rates slow down and solid substrates deplete, biofilm size was expected to continuously diminish while the planktonic fraction became predominant. Indeed, over 24 hours, the planktonic cell abundance increased 100 fold (Fig. S2) while the rate of cell accumulation (with a hypothetical t_gen_ = 0.82 h) greatly exceeded known growth rates^22^, demonstrating that the planktonic fraction increase primarily was due to continuous recruitment of cells from the biofilm populations at a pace that mirrored fermentation profiles (Fig. S2). Our novel bioreactor set-up will facilitate future biofilm ‘omics’ studies using different time points, lignocellulosic substrates, or different cellulolytic biofilm forming bacteria or consortia.

RNA-Seq analysis of 5.0 to 6.5 million paired-end sequencing reads per sample were aligned in CLC Genomics Workbench to the reference genome [GenBank:CP000568.1] with 3370 reference genes (Table S1). Sequencing depth was comparable across replicate planktonic and sessile samples with population averages of uniquely filtered and aligned gene reads (i.e., mapped fragments) of 2.11 (±0.10 std. dev.) and 2.04 (±0.09 std. dev.) million, respectively. Sequencing paired ends were counted as one gene read (i.e., one mapped fragment). Descriptive statistics of raw gene reads and their normalized FPKM (fragments per kilobase per million of mapped reads) were summarized in Tables S2 and S3. FPKM were used to estimate relative gene expression within a population. Differential gene expression analysis conducted with the R package DeSeq2^23^, was represented by fold-differences with positive log_2_ values for higher expression in sessile cells and negative log_2_ for higher expression in planktonic cells (Supplemetal dataset file 1). The significance cutoff was set at two-fold changes (1 < log_2_ < −1) and a 5% false discovery rate. RNA-Seq results were validated by RT-qPCR analysis of a subset of six genes and an R^2^ value of 0.998 confirmed the quality of data (Fig. S3).

**Figure 3:**
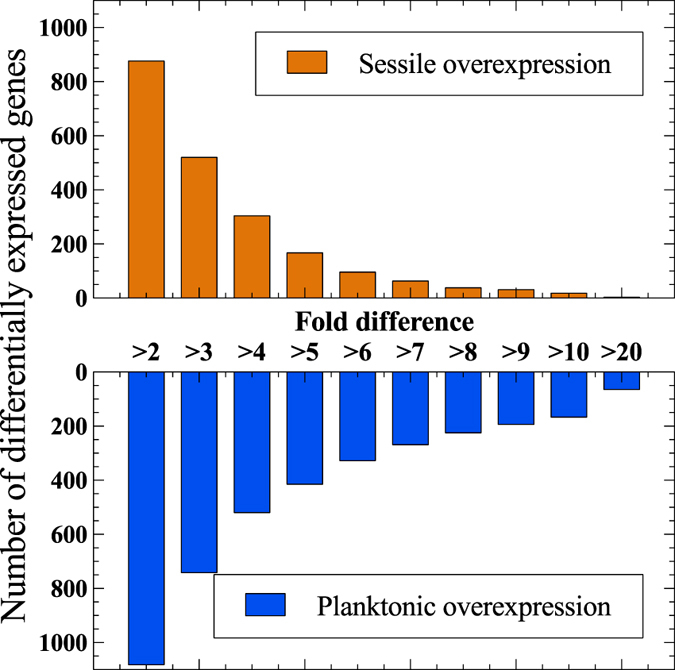
Number of differentially expressed genes grouped by minimum fold-differences of sessile (biofilm) compared to planktonic cell populations.

Of 3299 protein coding genes 71.4% (or 2356 genes) showed significant (p < 0.05) expression differences (with a minimum 1.22-fold change) across the two cell populations. A total of 1958 genes (or 59.3% of the protein coding genome) had a minimum two-fold differential expression between the two populations with 582 genes exhibiting at least a 5-fold change (Fig. 3), demonstrating the considerable degree of differentiation between the co-existent cell fractions.

To determine the metabolic pathways and functions most impacted by the genome-wide differential expression, genes were categorized based on current genome annotation, predicted functionality by association with ortholog groups available in the KEGG database^24^, in the organism-specific BioCyc database^25^, and based on literature evidence, where appropriate. Table 1 summarizes the functions and pathways with definitively higher gene expression in biofilms or planktonic cells discussed in detail in the following sections. The complete gene lists of these functions, their differential expression, the raw genome alignment reads and the FPKM are tabulated in Tables S1–S4; however, representative genes and their expression are specified in text.

Sessile biofilm cells (SS) had significantly higher expression of genes involved in sugar catabolism and energy generation, the anabolism of proteins and lipids, and in critical cellular functions that support cell division. Planktonic cells (PL) expressed genes for cell motility, nutrient sensing, the extracellular hydrolysis of polymeric sugars, and for a range of protection mechanisms such as oxidative stress response and repair, cell growth control, and recovery of damaged nucleic acids and proteins. This is unlike the classical multi-layer biofilm models whereby growth is dependent on flux-limited diffusion of nutrients or oxygen deep into the biofilm; and where transcriptomes of biofilms compared with their planktonic cell yields showed downregulated carbohydrate catabolism, protein and lipid anabolism and the overexpression of stress-related mechanisms for *Clostridium perfringens*^12^.

### Carbohydrate catabolism upregulation in biofilms

#### Central metabolism: cellodextrins to end-point metabolites and hydrogen metabolism

**Figure 4:**
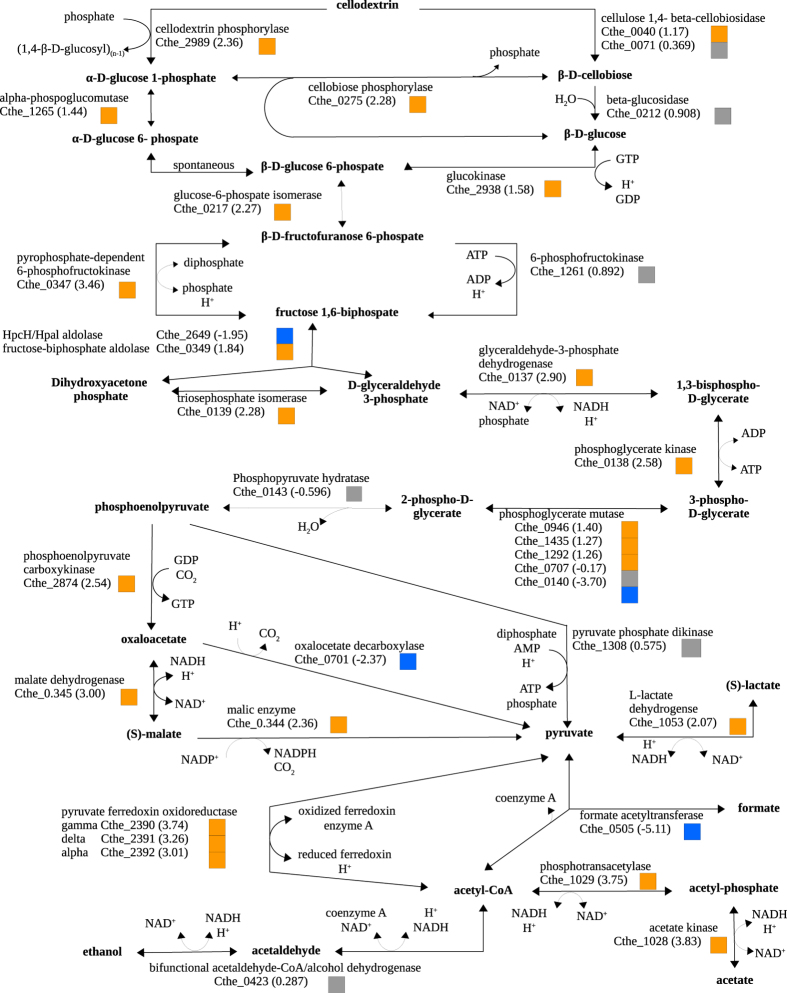
Central metabolism from intracellular cellodextrin sugars to end-point pyruvate fermentation products with gene annotation by product name and locus tag. Differential expression values in parentheses are represented by the logarithm (base two) of the fold-change in gene expression between biofilm and planktonic cell populations. Positive or negative values denote higher expression in biofilm or planktonic samples, respectively. Orange (higher in biofilm) and blue (higher in planktonic) color-codes denote minimum two-fold differential expression, while gray marks below two-fold differences.

Of the 34 genes involved in intracellular cellodextrins cleavage, glycolysis, and pyruvate fermentation (Fig. 4), 23 had a minimum two-fold overexpression in biofilms. Central metabolism was generally highly expressed in the SS genome where fourteen genes ranked in the top 5 percentile FPKM. Intracellular β-linked cellodextrins and cellobiose are converted to β-D-glucose-6-phosphate through hydrolysis (Cthe_0040, Cthe_0071, Cthe_0212) and GTP-consuming phosphorylation (Cthe_2938)^26^ or through phosphorylitic bond cleavage (Cthe_2989, Cthe_0275) without ATP consumption. Both paths showed increased gene expression in SS over PL; whereby higher transcription was observed for phosphorolytic rather than hydrolytic cleavage, indicating a preference for this energetically favorable route with reported higher *in-vitro* rates than hydrolysis^27^. Glycolysis to phosphoenolpyruvate had notably higher gene expression in biofilms. Of note, within this population, was the higher transcription of the pyrophosphate-dependent (Cthe_0347) versus the ATP-dependent (Cthe_1261) 6-phosphofructokinase, which substantiates two previous reports as the preferred route for the phosphorylation of β-D-fructofuranose 6-phosphate to fructose 1,6-bisphosphate^28^,^29^. The HpcH/Hpal aldolase (Cthe_2649) with differential expression in favor of PL (Fig. 4) belongs to a large operon (Cthe_2639–2649) with low transcription in either cell population and putative functions in sugar nucleotide biosynthesis, thus suggesting it is not a significant glycolytic gene. Comparatively, the fructose-biphosphate aldolase (Cthe_0349) ranked in the top 1 percentile of SS gene expression. *Clostridium thermocellum* has five putative phosphoglycerate mutase genes for the 3-phospho-D-glycerate conversion to 2-phospho-D-glycerate. One mutase (Cthe_0140) stood out, ranking in the top 5 percentile PL gene expression and nearly 13-fold higher than the SS equivalent. Considering the availability of homologous mutases with high transcription and the generally lower expression of central metabolism in planktonic populations, it is plausible that Cthe_0140 is not associated with the glycolytic pathway. The high-energy phosphoenolpyruvate (PEP) is converted to pyruvate by three mechanisms. In the absence of a known pyruvate kinase, the direct conversion is proposed^30^ to employ the pyruvate phosphate dikinase (Cthe_1308). Its deletion was shown to have little impact on pyruvate formation and the *in-vitro* activity on PEP was not detected^26^. While its role remains unknown we report this gene to have above average expression and only 48% higher expression in biofilms. The alternative route of PEP conversion via oxaloacetate with GTP production showed nearly six-fold higher PEP-carboxykinase expression in SS (a top 5 percentile gene). Oxaloacetate to pyruvate conversion showed very high expression of the malate dehydrogenase and the malic enzyme (Cthe_0345, Cthe_0344) in the malate shunt of sessile cells, while planktonic populations upregulated the oxaloacetate decarboxylase (Cthe_0701) route, a first indication of a potential NAD^+^/NADH imbalance.

Planktonic cells highly expressed the formate acetyltransferase gene (Cthe_0505 or *pfl*) for pyruvate conversion to acetyl-CoA with coproduction of formate, a metabolite detected via HPLC (Fig. 1). With over 34-fold overexpression, *pfl* and its activating enzyme gene (Cthe_0506) were the 12^th^ and 14^th^ most expressed genes in PL samples indicating this may be a key metabolic switch for carbon challenged and redox imbalanced cell populations. Biofilms employed the pyruvate ferredoxin oxidoreductase, *pfor*, with three gene subunits Cthe_2390–2932, which reduces a ferredoxin co-factor to generate the acetyl-CoA intermediate. In this case, reduced ferredoxin is re-oxidized in hydrogen metabolism by four hydrogenase systems which had significant overexpression in SS: the *hyd*-activated Fe-Fe hydrogenases (Cthe_0340–0342, Cthe_0428–0430 and Cthe_3003) and the Ni-Fe *ech* hydrogenase complex (Cthe_3020–3024)^31^. Drawing on the malate shunt example, it appears that biofilms utilized reactions that depended on stable redox cofactor recycling, while planktonic cells favored the alternative pathway, yet again indicating a redox imbalance. Acetyl-CoA fermentation to acetate via phosphotransacetylase (Cthe_1029) and acetate kinase (Cthe_1028) was >13-fold overexpressed in biofilms, while ethanol production through the bifunctional acetaldehyde-CoA/alcohol dehydrogenase gene (Cthe_0423 or *adhE*) did not show significant differential expression. The expression of *adhE* ranked in the top 5 percentile for SS and PL populations, and also highly expressed with little^21^ to no differential expression^4^ in other studies.

Hydrogen production in central metabolism by Fe-Fe and Ni-Fe hydrogenases consumes protons as final electron acceptors in the re-oxidation of ferredoxin and NADH^32^, which creates a gradient that powers proton influx over membrane F-type (Cthe_2602–2609) and V-type (Cthe_2262–2269) ATPases to generate ATP from ADP. The fourteen genes of these assemblies had 2.6- to 7.4-fold greater expression in biofilm cells suggesting higher productivity and energy yield in the adherent population. Co-expression of hydrogenases and H^+^-ATPases compares favorably to transcriptomic analysis of exponential growth cultures in a previous study^17^.

#### Nicotinamide adenine dinucleotide (NAD) biosynthesis

NAD+ and NADP+ are important co-factors in redox reactions where the former is usually involved in catabolism and the latter in anabolism. Genes for NAD+ biosynthesis via L-aspartate (Cthe_0325, Cthe_2355, Cthe_2356, Cthe_1241) as well as the ATP-NAD kinase (Cthe_0816) for NAD+ to NADP+ conversion had up to 6.5-fold higher expression in SS. This supports the assertion that biofilms have higher rates of carbohydrate catabolism and cell growth anabolism.

#### Oligosaccharides uptake by ABC transporters

Five putative sugar ABC transporters were previously identified in *C. thermocellum*, and their purified solute-binding proteins interact with various pentose and hexose sugars^33^. The *cbpC, cbpD* and *lbp* genes showed very low transcription levels, while the *cbpA* and *cbpB* transporters ranked in the top 5 or 1 percentile expression in both PL and SS populations. In particular, the solute-binding lipoprotein of CbpB with affinity for G2 to G5 cellodextrins was the most expressed gene in the SS genome, and its transcription unit (Cthe_1018–1020) had more than 2.5-fold greater expression compared to PL. We note that the PL-overexpressed CbpA and CbpD have reported ribose, xylose and autoinducer-2 general transport function inferred by sequence homology with RbsB in the KEGG database indicating a more promiscuous transport function. A sixth ABC transporter gene, Cthe_1862, containing the conserved ATPase domain of the maltose transporter MalK was highly expressed in biofilm cells. Our findings suggest that CbpA, CbpB and Cthe_1862 are the only highly expressed sugar transporters – an observation that is in line with previous transcriptomic^17^ and proteomic^28^ reports.

### Increased protein and lipid anabolism in biofilms

#### Amino acid metabolism

Genes for synthesis of 15 of the 20 primary amino acids were overexpressed in biofilms, and included: L-arginine, L-aspartate, L-asparagine, L-lysine, phenylalanine, L-tyrosine, L-histidine, L-valine, L-leucine, L-isoleucine, L-threonine, L-serine, glycine, L-glutamate and L-glutamine. We highlight the important branched-chain amino acid aminotransferase (Cthe_0856) necessary for valine, leucine and isoleucine biosynthesis with six-fold higher expression in SS. Biofilms had increased gene expression for *de novo* synthesis of L-glutamate via 2-oxoglutarate and ammonium and for L-glutamine via L-glutamate; however, planktonic cells overexpressed a six-gene operon (Cthe_0197–0202) with predicted L-glutamine to L-glutamate interconversion. In line with increased biosynthesis, two amino acid ABC transporters (Cthe_1456–1458 and Cthe_2278–2280) had 2.1- to 5.5-fold greater expression in biofilms. In *C. thermocellum*, amino acids are produced in large amounts at high cellulose loadings (e.g., 50 to 100 g/L Avicel) as overflow metabolites, where L-valine and L-alanine, for example, were secreted at up to 7.5 g/L concentrations^34^. Along with their primary role in anabolism, the amino acid carbon sink model supports the hypothesis of metabolically active carbohydrate-rich biofilms. Although L-valine genes are highly overexpressed in SS, we point out that a transaminase for L-alanine production from pyruvate or branched amino acids has not been identified in the reference genome. Alternatively, an L-cysteine desulfurase gene (Cthe_0655) that co-produces L-alanine and a protein sulfur acceptor from L-cysteine had two-fold higher expression in the PL population. Additionally, a putative serine-pyruvate aminotransferase (Cthe_0265) for production of L-alanine from L-serine was found by homology in a BLAST protein search, and had slightly higher expression in PL. L-cysteine is produced via L-serine by the serine *O*-acetyltransferase gene (Cthe_2066) which had over 15-fold higher expression in PL. Methionine is derived from L-cysteine and L-homoserine by three biosynthetic genes which also had significantly higher expression in PL. We therefore suggest that carbon deprived free cells increased production of sulfur-containing amino acids and sulfurated carriers (via L-alanine synthesis), which have a role in sulfur metabolism and the redox homeostasis of planktonic cells that will be discussed in following sections.

#### Ribosomal assembly

Within 54 ribosomal protein genes, 50 showed two- to fourteen-fold higher expression in SS cells consistent with greater metabolic activity. All genes were generally well represented in the SS transcriptome, where 40 ranked in its top 5 percentile. Methyltransferase and maturation factor genes responsible for ribosomal protein modifications and for the ribosomal RNA assembly were overexpressed in SS as well.

#### Charging of tRNA

Aminoacyl-tRNA synthases bind amino acids to their compatible accepting tRNA molecules in preparation for ribosomal translation. Genes for 17 of the 20 synthases had minimum two-fold higher expression in SS cells to support increased ribosomal activity, while only cysteine-tRNA synthase had 13.5-fold higher expression in PL cells, indicating a necessity for cysteine-rich proteins such as di-sulfur bridge or Fe-S cluster redox enzymes as discussed more below.

#### Translation initiation and elongation

Six of eight initiation and elongation factors were greater than 3.3-fold overexpressed in biofilms and in particular elongation factor Tu (Cthe_2930) showed very high transcription. The SmpB (small protein B) gene (Cthe_2748), essential for the translational surveillance and ribosomal rescue function of the SsrA system, had nearly three-fold higher expression in SS.

#### Chaperone

Like molecules which assist in the correct folding and assembly of polypeptides, specifically, chaperones Hsp70 (Cthe_1321–1323)^35^, Hsp33 (Cthe_1852) and GroES/EL (Cthe_2891 and Cthe_2892) were all overexpressed in SS. Also important in correct protein folding, cyclophilin Cthe_0068 had four-fold higher expression in SS.

#### Polyamines

Putrescine and spermidine, growth-essential small polycationic molecules that associate with DNA to control the rate and fidelity of protein synthesis, can be produced *de novo* from L-arginine as well as imported and exported from the cell by an ABC-type transporter^36^,^37^. The synthesis genes to some extent, and in particular the transporter genes had higher expression in biofilms.

#### Protein secretion and maturation

The Sec secretion system is a general mechanism employed by gram-positive bacteria for protein translocation across or insertion into the cytoplasmic membrane^38^. The signal recognition particle (Cthe_0770) binds the signal sequence of ribosome-associated pre-proteins and interacts with its membrane receptor (Cthe_0926) to bring the protein to the translocase motor SecA (Cthe_1385) and the protein conducting channel SecYEG (Cthe_2923, Cthe_2718 and Cthe_0144) with its accessory proteins Cthe_0904, Cthe_0903 and Cthe_0957 for translocation outside the cell. Insertion in the cytoplasmic membrane is mediated by YidC (Cthe_2367). Membrane-bound signal peptidases (Cthe_0350, Cthe_0764, Cthe_0909, Cthe_1330, Cthe_1421 and Cthe_2079) cleave the signal sequence after translocation. These genes had significantly higher expression in biofilms, which adds further evidence to the overall increased protein anabolism in the cellulose-adherent cell fraction.

#### Fatty acids and other cell wall components

Fatty acid initiation and elongation and glycerophospholipid metabolism, in particular the synthesis of phosphatidylglycerol and CDP-diacylglycerol, are required for cytoplasmic membrane renewal and cell division, while peptidoglycan synthesis is needed to maintain cell shape and osmotic stability. Genes for fatty acid and phospholipids synthesis all exhibited significantly increased expression in biofilms. Peptidoglycan genes were well represented by moderate gene expression in both populations, and in particular we observed the PL overexpression of the *mraZ* operon (Cthe_0974–0980), containing five peptidoglycan-synthesis specific genes which co-express with two spore-cortex formation genes. The bacterial S-layer proteins form a monomolecular layer on the cell envelope (i.e., the peptidoglycan) and support the binding of surface proteins (e.g., the cellulosomal anchorin scaffoldins) and other specific anchoring ligands that mediate swimming, aggregation, and biofilm formation. S-layer proteins have also a presumed role in bacteriophage and bacteriocin resistance, maintenance of cell envelope integrity and in selective permeability^39^. We report expression levels of non-cellulosomal S-layer homology (SLH) domain-containing genes (Table S4). From these, we highlight Cthe_1368 with high expression in both populations; a gene with no other conserved domains which we suggest may represent the primary S-layer protein of *C. thermocellum*. Additionally, a second S-layer gene (Cthe_2506) with 25-fold higher expression in PL contained conserved laminin, cadherin and cellulose-binding domains that have primary functions in cell-adhesion, implying a role in biofilm initiation.

### Cell proliferation support functions overexpressed in biofilms

#### Cell division proteins

Genes of the membrane associated proteins involved in cell division and septation, FtsY/X/E (Cthe_1858–1860) and FtsQ/A/Z (Cthe_0442, Cthe_0444–0445) had significantly higher expression in biofilms. Two proteins associated with spore cortex formation and spore septation in *Bacillus* spp., FtsW/SpoVE (Cthe_0975) and FtsL (Cthe_0980), belong to the *mraZ* operon^40^ and had higher expression in PL. The FtsK/SpoIIIE DNA translocase membrane protein complex is involved in chromosome segregation into dividing daughter cells or into the forespore^41^. Of its four homologues, we highlight Cthe_1095 with 8-fold greater expression in PL cells suggesting a possible involvement in forespore formation, when considering the general overexpression of sporulation genes in PL.

#### Purines and pyrimidines

Fast metabolism, cell growth, and division was supported and accompanied by increased *de novo* synthesis of ribonucleotides and deoxyribonucleotides. To that end, 28 of the 30 genes involved in pyrimidine ribonucleotide (UTP, CTP) and deoxyribonucleotide (dCTP, dTTP) *de novo* biosynthesis via L-glutamine and L-aspartate and of purine ribonucleotide (ATP, GTP) and deoxyribnucleotide (dATP, dGTP) *de novo* biosynthesis via L-glycine, 5-phosphoribosyl pyrophosphate (PRPP) and L-aspartate were significantly overexpressed in the SS population by up to 10.9-fold.

#### Biosynthesis of folates

Tetrahydrofolates (THF), which function as one-carbon group donors, are essential cofactors for *de novo* synthesis of thymidine monophosphate, an integral component of nucleic acid metabolism and therefore, of normal cell growth and replication. Genes of THF synthesis from GTP and its transformations to methenyl- and methyl-THF forms had higher expression in SS.

#### Homologous recombination

Genes for the RecFOR, RuvABC and the RecD of the RecBCD (RecB and RecC missing annotation) homologous recombination systems, were generally upregulated in the SS population, although with only average expression levels. DNA polymerases, were not greatly differentially expressed.

### Motility, chemotaxis and nutrient scavenging upregulated in planktonic cells

#### Chemotaxis

The bacterial response to changing concentrations of external chemicals is directed towards finding higher levels of a required nutrient source or fleeing a stressor. Although chemotaxis gene transcription was generally not high, the PL population had a notable greater expression of 20 of the 31 putative genes involved in this process, which included membrane sensory transducers and intracellular methyl transferases and esterases, pointing to the planktonic cell adaptation to the low carbon environment and potential stress sources.

#### Cell motility

Part of a two-component system, chemotaxis is coupled with cell motility by flagellar swimming or pili twitching^42^. Of the 43 putative genes involved in flagellar assembly and motility, 35 showed significantly greater expression in PL cells. Type IV pilus proteins PilZ, PilM and PilT involved in the assembly and retraction motility of bacterial pili were found to have higher expression in PL cells where 10 out of the 16 putative pili genes had greater than two-fold expression, compared to only 1 gene for the SS population.

#### The cellulosome and free cellulases

The *C. thermocellum* genome contains genes for 69 cellulosomal cellulases, 8 non-catalytic cellulosomal proteins and 27 non-cellulosomal free cellulases^4^. We noted 50 cellulosomal cellulases (i.e., 72%) with significantly higher expression in the carbon deprived planktonic populations, of which 29 (i.e., 42%) had minimum 2-fold differential expression. Four genes (in decreasing order for: CelS, CtMan5A, CelK and CbhA) had remarkably high expression in both cell populations, and showed differential expression in favor of PL by at least 44% with statistical significance. CelA, CelJ and XynC also had generally high expression, with the latter two genes, a xyloglucan-specific exoglucanase and a beta-xylanase, being better represented in the sessile population, suggesting a readiness to deal with more complex xyloglucan substrates in the solid-attached cells. The primary cellulosomal scaffold, CipA (Cthe_3077), a large gene with the highest sequencing read counts and top 1 percentile FPKM in both populations, was not differentially expressed, which is supportive of its common use as reference gene in qPCR analyses. Cell-anchoring scaffoldins with very high expression that mediate cell-envelope binding of cellulosomes (type II cohesins OlpB and Orf2p) or non-cellulosomal cellulases (type I cohesin OlpA), had a minimum 3-fold higher PL expression. The mobile ScaE cellulosomal scaffold, suspected to form cell-detached cellulosomes^4^, had a significant, though modest 40% increase in expression in SS. Differential expression of free cellulases was evenly divided between the two populations where each cell fraction had 9 genes with minimum 2-fold higher expression; however, biofilms showed better expression of the genes with above average FPKM. It is concluded that carbon limited planktonic cells increased the expression of surface-associated cellulosomes to enhance carbohydrate solubilization and carbohydrate binding domain-mediated cellulose adhesion, while cellulose-attached biofilms favored the mobile cellulosomes and free cellulases.

### Oxidative stress response in the planktonic population

#### Antioxidants

Planktonic cells showed remarkably high expression of antioxidant proteins with di-sulfur bridges or iron-sulfur cluster cores. In particular, Cthe_0360, Cthe_1965, Cthe_1964 and Cthe_0063 were the most expressed genes in the PL population, with a minimum 31-fold higher expression over biofilms. For example, thioredoxin (Cthe_0360) with 36.7-fold higher PL expression maintains redox homeostasis by reducing oxidized proteins through the reversible conversion of two cysteine thiol groups to a disulphide. Related to this protein, and with similar antioxidant function, are glutaredoxin, peroxiredoxin, alkyl hydroperoxide reductases and FAD-dependent pyridine nucleotide-disulphide oxidoreductases which were all highly overexpressed in planktonic cells. Rubredoxin-like Fe(Cys)4 protein genes (Cthe_0063 and Cthe_2164) had up to 33.4-fold higher expression in the stress-responding PL population. This class of proteins has the most basic Fe-S cluster-bearing structure where the iron atom is ligated by four cysteine residues and is strictly found in anaerobic bacteria and archaea where it participates in the reduction of oxygen and peroxy species with other redox partner proteins. In *Desulfovibrio vulgaris* rubredoxin with *in vivo* oxygen oxidoreductase functions, was shown to enhance the cell survival in microaerophilic conditions as the anaerobic bacterium has evolved to withstand transient and periodic oxygen exposure in their natural environment^43^. The poorly annotated Cthe_1948 gene, with good amino acid sequence similarity to CcdA in *B. subtilis* and DsbD in *E. coli*, had 9-fold higher expression in PL, and it may be involved with transferring reducing equivalents across the cytoplasmic membrane and with efficient spore formation^44^. Interestingly, genes for the putative sequestration and transport of the copper ion (Cthe_0738 and Cthe_1848), a cation often found in electron transfer metalloproteins, were also greatly upregulated and over 18-fold overexpressed in the PL population. Lastly, a hemerythrin/HHE cation binding domain protein (Cthe_1509) from a family of oxygen-scavenging proteins was found to have over 70-fold higher expression in PL cells where it ranked in the top 5 percentile. The peptide methionine sulfoxide reductase (MsrA) (Cthe_2990) responsible for the repair of the most redox-sensitive amino acid, methionine, also showed over 15-fold higher expression in planktonic cells.

#### Flavoprotein biosynthesis

Flavin adenine dinucleotide (FAD) and flavin mononucleotide (FMN) are co-factors utilized in energetically difficult oxidation reactions and are required for the antioxidant functions described above. Genes for their biosynthesis via riboflavin were unsurprisingly significantly upregulated in PL.

#### Iron-sulfur clusters

Fe-S clusters in proteins have the essential primary role of electron transfer in cellular reactions where they operate at a wide range of physiologically relevant redox potentials^45^. As iron and sulfur species can be reactive and toxic *in vivo*, the synthesis of Fe-S clusters is coordinated by specialized systems in prokaryotes. The two adjacent genes of this Fe-S cluster assembly and repair mechanism are the cysteine desulfurase NifS (Cthe_0720) and the U-type scaffold NifU (Cthe_0721), both highly overexpressed, over 24-fold, in PL where they ranked in the top 1 and 5 percentile FPKM, respectively. Oxidation causes Fe-S cluster damage, therefore increased *de novo* synthesis is commonly reported to be upregulated in oxidative stress and mediated by various redox stress transcriptional factors^45^.

#### Sulfate and Iron(III) uptake and the sulfur metabolism

To support increased Fe-S cluster synthesis and the generalized oxidative stress response, free cells also increased expression of ABC transporter genes for sulfate and Fe(III) uptake, although with modest transcription. Similarly, genes for assimilatory sulfate reduction to sulfite and the genes in the siroheme biosynthetic pathway, which converts sulfite to biologically relevant sulfide, were better represented in PL cells. This evidence conforms to previous reports where the opposite transcriptional response, i.e., the downregulation of sulfate upkate, assimilation and of siroheme genes, was recorded after further reduction of the growth medium from steady-state conditions^46^. The combined evidence distinctly illustrates a planktonic population that activated an array of mechanisms best described as induced by redox perturbations.

### Stress induced homeostasis and repair in planktonic cells

#### tRNA thiolation

Interestingly, downstream and in the same transcription unit with the iron-sulfur cluster genes, a tRNA-specific 2-thiouridylase (Cthe_0722) gene was found with nearly 17-fold increased expression in PL. It is responsible for wobble uridine thiolation in tRNA, important for the translation of growth-specific proteins (rich in lysine, glutamine and glutamate codons) and suggested to be involved in the regulation of translational capacity and metabolic homeostasis in nutrient challenging conditions^47^, where thiolation provided cells a strong growth advantage against thiolation-deficient mutants. The same study suggests that reduced tRNA thiolation resulted in the reduced production of sugar metabolism proteins and it concludes that availability of sulfur amino acids controls tRNA thiolation. The increased PL overexpression of L-cysteine and methionine synthesis genes may therefore be linked to the upregulation of iron-sulfur cluster genes not only for Fe-S protein assembly, but also for translational control of cell growth under nutrient stress. The literature presents numerous other examples of fast-tracking the production of key proteins under damage, stress and disease conditions controlled by tRNA modifications of wobble bases, where the mRNA of desired stress response proteins have significant bias in the use of degenerate codons that have optimal binding to the modified tRNA^48^.

#### Indole synthesis

A five-gene operon (Cthe_0871–0875) with high expression and upregulation in PL cells, encodes the metabolic pathway for indole production via chorismate. All five genes ranked in the top 5 percentile of PL expression, pointing to the important role for this metabolite. Indole production and secretion greatly increased in *E. coli* under limited nutrient conditions where it stimulated phage-shock responses and oxidative stress responses^49^. At least 85 bacterial species are known to produce large quantities of indole where it is involved in quorum sensing, cell cycle regulation, acid resistance, and species-dependent positive or negative signaling of biofilm formation^50^. It has been proposed that this novel signaling molecule triggers protective responses that prepare a cell population against future stress. To our knowledge, this is the first account of the possible roles of indole in this organism, and we observed excellent conformity between literature reports and the current proposed model of nutrient-challenged planktonic populations that activated comprehensive protective mechanisms.

#### Nucleotide excision repair (NER) system

Random structural anomalies in DNA molecules occur through exogenous damage due to environmental factors such as UV light or through endogenous damage such as lesions cause by reactive oxygen species. As sessile and planktonic cells were subject to similar environmental stress in the well mixed reactors, endogenous damage by oxidative stress may explain differential expression of the primary DNA repair mechanism, the nucleotide excision repair system. Genes of the UvrABC excinuclease complex (Cthe_0311, Cthe_0309, Cthe_2737) and the UvrD helicase (Cthe_0206) had high transcription levels and up to 41-fold (e.g., UvrC) greater expression in PL cells.

#### Proteases and peptidases

Consistent with previous observations, protein damage occurs during periods of DNA damage and developmental changes induced by stress. Two of the better studied ATP-dependent protease systems, the LON proteases (Cthe_0082, Cthe_2742) involved in selective degradation of abnormal proteins^51^ and the CLP proteases (Cthe_1216–1217, Cthe_2740–2741, Cthe_1789, Cthe_0312) involved in the degradation of protein aggregates or terminally damaged polypeptides^52^ were highly expressed in PL samples. For example, four of the eight representative genes ranked in the PL top 5 percentile and had 8- to 26-fold higher expression over biofilms. Similarly, the ATP-dependent metalloprotease FtsH (Cthe_2253) with higher expression in PL (top 5 percentile) is responsible for the disassembly of misfolded or damaged membrane proteins.

#### Sporulation

Of the 77 putative genes associated with sporulation proteins, 51 had greater than 2-fold expression in PL cells while only 4 did in SS samples. Transcriptional control of sporulation is governed by the response regulator *spo0A* (Cthe_0812, Cthe_3087) which was not differentially expressed, however the putative sigma factors (Cthe_0447 and Cthe_0448) it potentially acts upon, had higher expression for PL. Spo0A is activated by post-translational phosphorylation; however, the putative histidine kinases (Cthe_2695, Cthe_2076 and Cthe_0286 with positive regulation and Cthe_0256 with negative regulation) proposed to control Spo0A phosphorylation^53^ did not show a consistent differential expression trend. Still, it must be noted that signal transduction mechanisms are poorly for this bacterium and the histidine kinase with the highest transcription, Cthe_2076, was 40-fold overexpressed in planktonic samples.

#### Phage associated proteins

The *C. thermocellum* genome was predicted to contain five intact prophage genomic regions^54^, covering loci Cthe_1608–1730, Cthe_1982–2021 and Cthe_2451–2501. Although, these coding sequences had generally low read counts, they were overall decidedly more highly expressed in PL, in particular for the latter genomic region with bacteriophage OH2 similarity. Literature evidence suggests the possibility of temperate phages that activate prophage expression and lysogenic conversion only during host stress^55^.

### Proteome and transcriptome comparisons

A proteomic analysis of sessile and planktonic fractions collected simultaneously with RNA-Seq samples was represented by 1406 gene products (Supplemental dataset file 2) or 43% of the protein coding genome. The extracellular cellulosome and free cellulase secretome was found predominantly immobilized on solid cellulose, for which it has specific binding affinity, often irreversible^56^, through cellulose binding domains. It is thus conceivable that detaching cells may leave cellulases bound to the solid substrate. Therefore, these products were excluded from comparisons between cellular proteomes and transcriptomes. Of the 1406 proteins identified across both cell populations (n = 4), 282 products (excluding cellulosomal proteins) showed significant (p < 0.05) abundance differences between the two populations with 70% of them correlated to the observed transcriptional differences. Imposing a minimum threshold of two-fold (log2 > 1) and significant (p < 0.05) transcription differences, the percent match with proteome data increased to 75%, which was in line with typical expectations reported in the literature^57^ and is likely due to fundamental differences between the two macromolecules including: temporal delays between mRNA generation and protein translation and maturation, their respective half-lives, as well as additional post-transcriptional and translational regulation mechanisms. Confirming that proteomic differences observed between the two populations required additional time to manifest, at least with respect to their relationship to gene expression, Pearson correlations between sessile and planktonic protein abundances were found to be increasingly divergent (Fig. S4) when the planktonic fraction was sampled at three additional later time points (up to 23 hours) (Supplemental dataset file 2). For example, ribosomal proteins were found to have similar abundances between both SS and PL at t = 12 hours (when the planktonic cell population was in early development), but with a marked decrease in abundance over time in PL cells – an observation that points not only to slower anabolism, as expected and suggested by RNA-Seq data, but also that it takes time for protein signals to manifest relative to what is inferred by gene transcription. Overall, the key functional differences listed in Table 1 were supported by proteomic analyses to the extent of the achieved proteome coverage.

In conclusion, we established that cellulolytic bacterial cells that evolved to thrive on solid carbon sources will thoroughly alter the genomic expression of their central metabolism, anabolism and homeostatic functions in response to the availability of solid attachment interfaces and solubilizable carbohydrates. The attached bacteria are functionally strong in growth and biomass conversion, while the planktonic cells are stressed and motile due to low available substrates. The specific physiological adaptations identified in this study may extend in part to a larger set of anaerobic, cellulolytic thermophiles that are under scrutiny for use in biofuel technologies. Future studies, therefore, have to carefully resolve the partitioning of adherent and non-adherent cells when using gene expression analysis to infer cellular functions, feedback or regulation.

## Materials and Methods

### Strains and culture conditions

*Clostridium (Ruminiclostridium) thermocellum* ATCC 27405 was maintained in 20% glycerol stocks and grown in standard MTC medium as described previously^58^.

### Batch bioreactors fermentations and isolation of cell fractions

**Figure 5:**
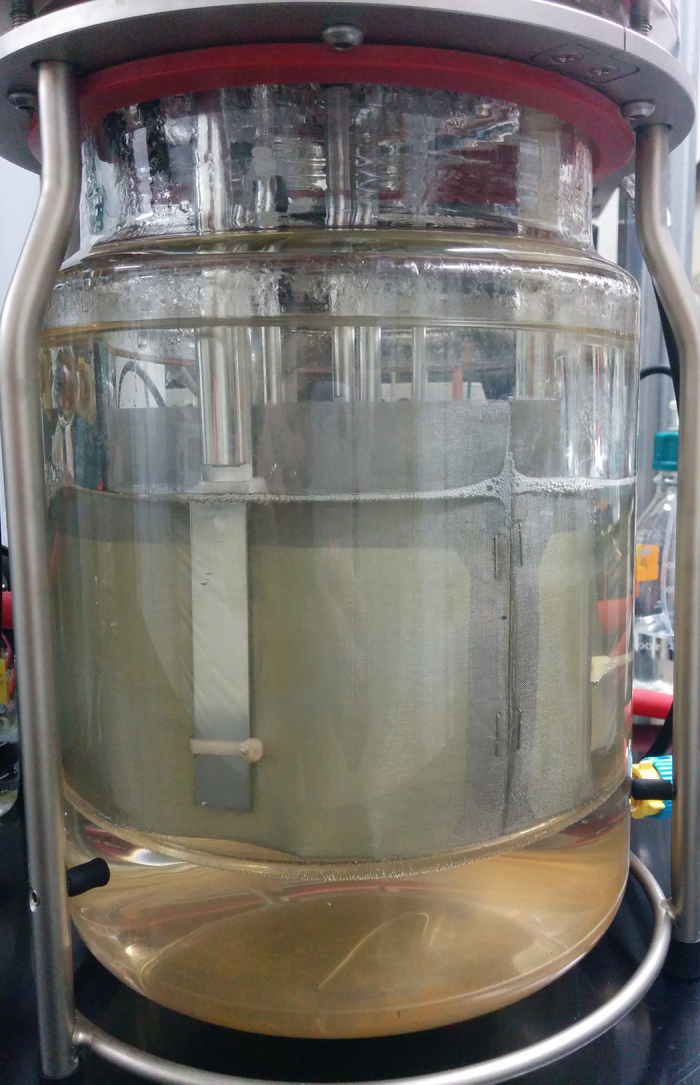
Bioreactor with stainless steel meshes adapted to baffles that support cellulose and biofilms, and were used for quick collection of sessile cells for RNA-Seq analysis.

Six replicate batch fermentations in MTC medium with Whatman #3 cellulose paper at 3 g/L as the sole carbon source were carried in 3 L Applikon bioreactors (Applikon Biotechnology, CA) with a 2 L fermentation volume at a constant pH of 7.00, 60.0 °C and 250 rpm impeller speed. Nitrogen gas at 2 mL/min was passed through the reactors and pH was adjusted by titration with 2 N potassium hydroxide. Reactors were inoculated (5% v/v) with fresh, overnight cultures. Four fermentations were ended 14 hours after inoculation with biofilm and planktonic cell fractions collected for gene expression and proteomic studies. Two fermentations were run 35 hours to completion for quantitative analysis of the fermentation process and time-course measurements of planktonic cells accumulation in the liquid medium. Three pockets made of stainless steel mesh (Spectrum Laboratories Inc., CA) with 213 μm pore size were tied to each reactor’s baffles (Fig. 5 and Fig. S5), and each housed a sheet of cellulose Whatman #3 paper for easy and fast collection of the biofilm (sessile) cell fraction. For RNA-Seq and proteomic analyses, the steel meshes housing cellulose and biofilm were first briefly rinsed in saline solution (0.9% NaCl) before flash freezing in liquid nitrogen and the frozen material was then stored at −80 °C in plastic conical tubes. The planktonic cell fraction was collected from the aqueous medium in 40 mL aliquots and drawn through a 20 μm nylon woven net filter (EMD Millipore, MA) to prevent contamination from cellulose particulates. After centrifugation at 9000 × g for 5 minutes at 4 °C, the supernatant was discarded and tubes containing cell pellets were flash frozen in liquid nitrogen and stored at −80 °C.

### Fermentation products and cell fractions quantification

Fermentation product formation and soluble carbohydrate content were measured by HPLC as described^58^. Planktonic and sessile cell fraction abundances at the RNA-Seq sampling time point were determined by total protein quantification using the BCA assay (Thermo Scientific) with prior purification of matrix interferences by trichloroacetic acid (TCA)-mediated precipitation^59^. Cell protein content (μg/mL) was correlated to direct cell counts (cells/mL) obtained from a separate set of time-course samples collected from the planktonic fraction during complete fermentations, and processed similarly. Cell enumeration by microscopy direct counting was performed as described in ref. 8.

### RNA isolation and ribosomal RNA removal

Trizol (Invitrogen, CA) was added to each sample and the mixture was lysed by bead beating with 0.8 g of 0.1 mm glass beads (BioSpec Products, OK) in a Retsch Tissue Lyser II (Retsch GmbH, Germany) for 3 × 20 s each at 25 Hz with a 1 minute pause between each disruption. RNA was prepared from each sample, cleaned on and RNeasy column, with on column DNaseI-treatment, and quantity and quality assessed, as previously described^18^. High quality total RNA (RIN > 8.4) was depleted of rRNA using Ribo-Zero rRNA Removal Kit for bacteria (Epicentre-Illumina, CA) following kit protocol. The depleted sample was purified on a RNA Clean & Concentrator-5 (Zymo Research, CA) following kit protocol.

### Library preparation, sequencing and RNA-Seq analysis

Depleted RNA was used for RNA-Seq library preparation with the Epicentre ScriptSeq v2 RNA-Seq Library Preparation Kit (Epicentre-Illumina, CA) following the manufacturer’s protocol (EPILIT329 Rev. C). Agencount AMPure beads (Beckman Coulter, IN) were used to purify the cDNA, and unique indexes were added during 13 cycles of library amplification. The final RNA-Seq libraries were purified with Agencount AMPure beads (Beckman Coulter, IN) and quantified with a Qubit fluorometer (Life Technologies, CA). The library quality was assessed on a Bioanalyzer DNA 7500 DNA Chip (Agilent, CA), and samples were pooled and diluted. Two paired end sequencing runs were completed on an Illumina MiSeq Instrument (Illumina, CA) using the standard protocol. Raw sequencing fragment reads were mapped in CLC Genomics Workbench 9.0.1 (CLC bio, Denmark) using default prokaryote settings to genome [GenBank:CP000568.1] and the resulting unique gene read counts output was used for differential expression analysis with the R package DeSeq2 according to published methodology^23^. RNA-Seq gene expression as well as raw sequencing and mapped reads data have been deposited in NCBI GEO accession number GSE87407.

### Proteomic analysis

Samples of planktonic and sessile fractions were re-suspended and homogenized in 3% SDS-PBS buffer by bead-beating at 25 Hz in 4 cycles of 20 seconds. Crude lysates were digested to peptides using trypsin to prepare for 2D LC-MS/MS analysis^59^. Twenty-five micrograms of tryptic peptides were then loaded onto a biphasic MudPIT back column (reversed phase [RP; 5 micron Kinetex, Phenomenex] and strong cation exchange [SCX; 5 micron, Luna, Phenomenex] resins), desalted, placed in line with an in-house pulled 100 micron ID nanospray emitter, and analyzed over 11 ammonium acetate salt cuts (120 min RP separation per cut) using an Orbitrap Pro (Thermo Scientific) mass spectrometer operating in data dependent acquisition. The resulting MS/MS spectra were searched against the *C. thermocellum* ATCC 27405 proteome database – concatenated with common contaminants and reversed protein entries to assess false-discovery rates (FDR) – using Myrimatch v. 2.1^60^. Peptide spectrum matches were filtered by IDPicker v.3^61^ (FDR < 1%), assigned matched-ion intensities, and peptide abundance distributions normalized and assembled to proteins using InfernoRDN, as previously described^4^. For statistical analyses, missing protein abundance values were first imputed with a distribution of values near the limit of detection and one way ANOVA performed using Perseus^62^ proteomics software.

## Additional Information

**How to cite this article**: Dumitrache, A. *et al*. Specialized activities and expression differences for *Clostridium thermocellum* biofilm and planktonic cells. *Sci. Rep.* **7**, 43583; doi: 10.1038/srep43583 (2017).

**Publisher's note:** Springer Nature remains neutral with regard to jurisdictional claims in published maps and institutional affiliations.

## Supplementary Material

Supplementary Dataset 1

Supplementary Dataset 2

Supplementary Information

## Figures and Tables

**Table 1:** Summary of synthesis pathways and cellular functions with notable and significant higher gene expression (minimum two-fold) in the sessile (biofilm) or planktonic cell populations.

Sessile (SS)	Planktonic (PL)
**Catabolism (central metabolism)**	**Motility, nutrient sensing & scavenging**
Intracellular cellodextrin glycolysis and fermentation	Chemotaxis
Hydrogen metabolism with four hydrogenase systems	Flagellar & pili assembly
ATP regeneration by proton-dependent ATPases	Cellulosomal cellulases and anchoring scaffoldins
NAD/NADP *de novo* biosynthesis	
	**Oxidative stress response**
**Anabolism (protein and lipid synthesis)**	Antioxidants (e.g., thioredoxins, peroxiredoxins)
Biosynthesis for 15 of the 20 primary amino acids	Flavoproteins (FAD, FMN) – antioxidant co-factors
Ribosomal protein synthesis and the tRNA charging	Methionine repair by MsrA
Translation initiation and elongation factors	Fe-S cluster assembly and repair for redox proteins
Chaperones for correct protein folding	Iron and sulfate uptake and assimilatory reduction
Protein secretion and maturation (‘Sec’ pathway)	Sulfur amino acids biosynthesis
Polyamines for fidelity control of protein synthesis	
Fatty acids initiation/elongation & glycerophospholipids	**Stress induced cellular homeostasis and repair**
	tRNA thiolation for regulation at translation level
**Cell proliferation**	Indole synthesis for a variety of cellular stress responses
Cell division and septation proteins	Sporulation proteins
*De novo* synthesis of purine and pyrimidine nucleotides	DNA damage repair by nucleotide excision repair
Biosynthesis of folates for one-carbon metabolism	Removal of damaged proteins by LON and CLP proteases
Homologous recombination (RecFOR and RuvABC)	
