# Supplementary material for: Specialized activities and expression differences for Clostridium thermocellum biofilm and planktonic cells
Source: Sci Rep. 2017 Feb 27;7:43583. doi: 10.1038/srep43583 (PMC5327387; doi:10.1038/srep43583)
Supplement: Supplementary Information [file srep43583-s3.pdf]

Title: Specialized activities and expression differences for *Clostridium thermocellum* biofilm and planktonic cells

Authors: Alexandru Dumitrache[1,2] (dumitrachea@ornl.gov), Dawn M. Klingeman[1,2] (klingemandm@ornl.gov), Jace Natzke[1,2] (natzkejm@gmail.com), Miguel Rodriguez[1,2] (rodriguezmjr@ornl.gov), Richard J. Giannone[1,3] (giannonerj@ornl.gov), Robert L. Hettich[1,3] (hettichrl@ornl.gov), Brian H. Davison[1,2] (davisonbh@ornl.gov), and Steven D. Brown[1,2] (brownsd@ornl.gov)

Supplementary Information

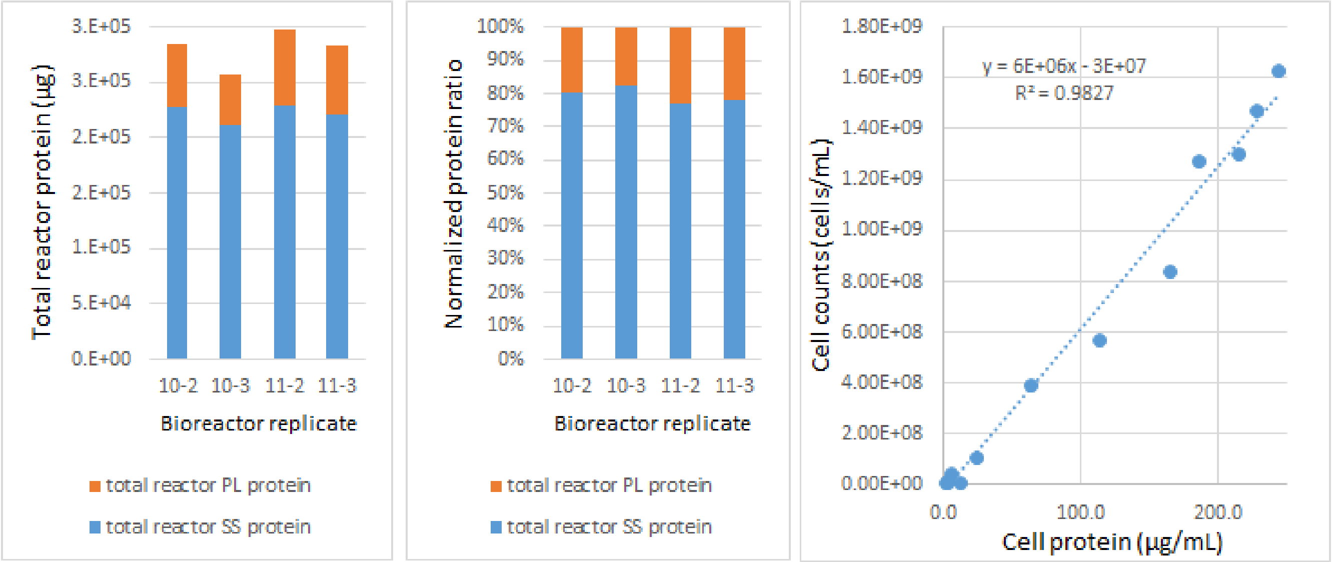

Fig S1. Sessile and planktonic total cell protein (µg) content at the time of RNAseq collection (LEFT); the normalized % ratio between planktonic and sessile protein content at the time of RNAseq collection (MIDDLE); the correlation between cell protein measurement (µg/mL) and cell count enumeration (cells/mL) (RIGHT).

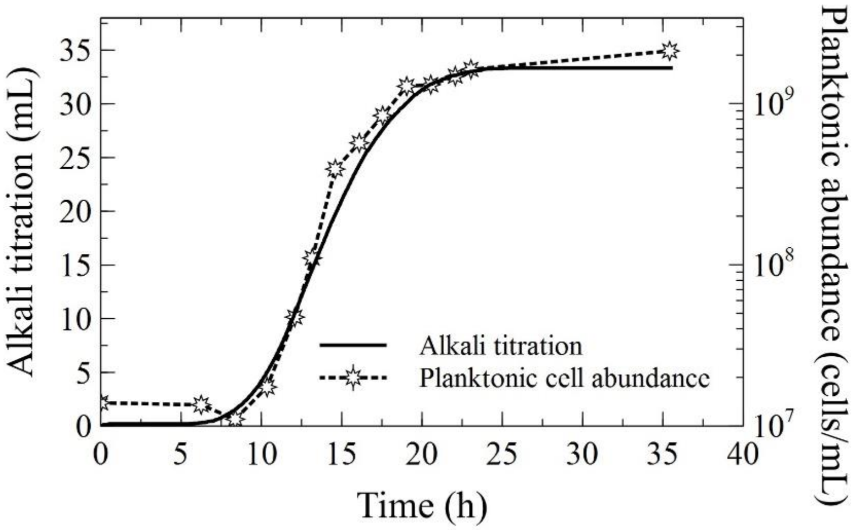

Fig. S2. Time-course measurement of planktonic cell abundance showed free cell accumulation that matched the rate of metabolite accumulation (shown as the alkali titration curve). Averaged data of biological duplicate fermentations.

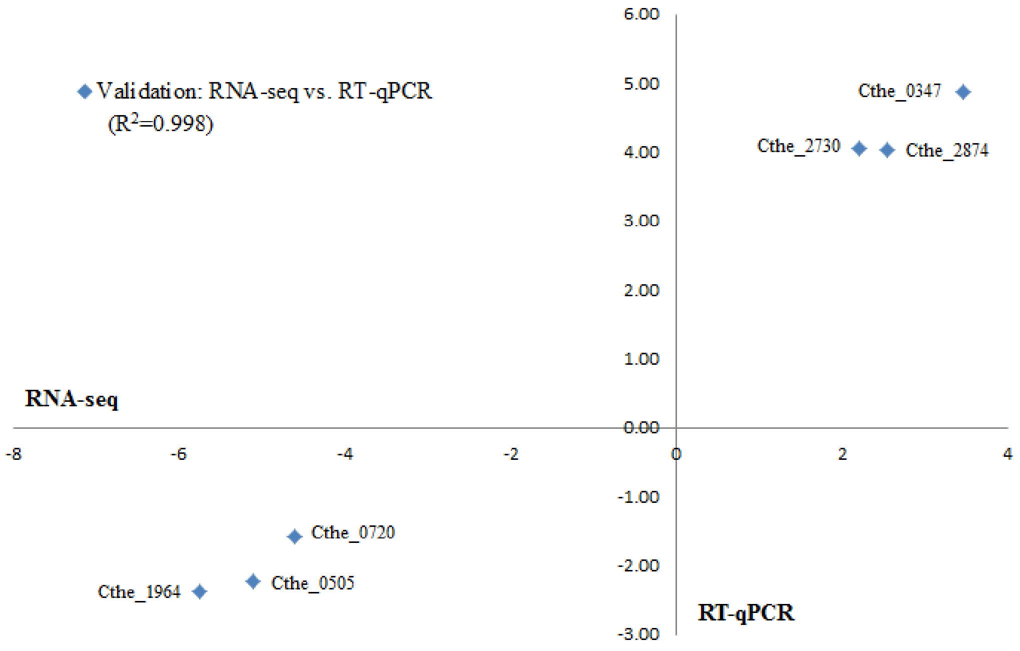

Fig.S3. RT-qPCR validation of RNA-seq expression. The log_2-transformed fold changes in a subset of six genes were compared by RT-qPCR and RNA-seq. Linear regression variance (R^2) of 0.998 confirmed a good correlation between the two analyses confirming the quality of data.

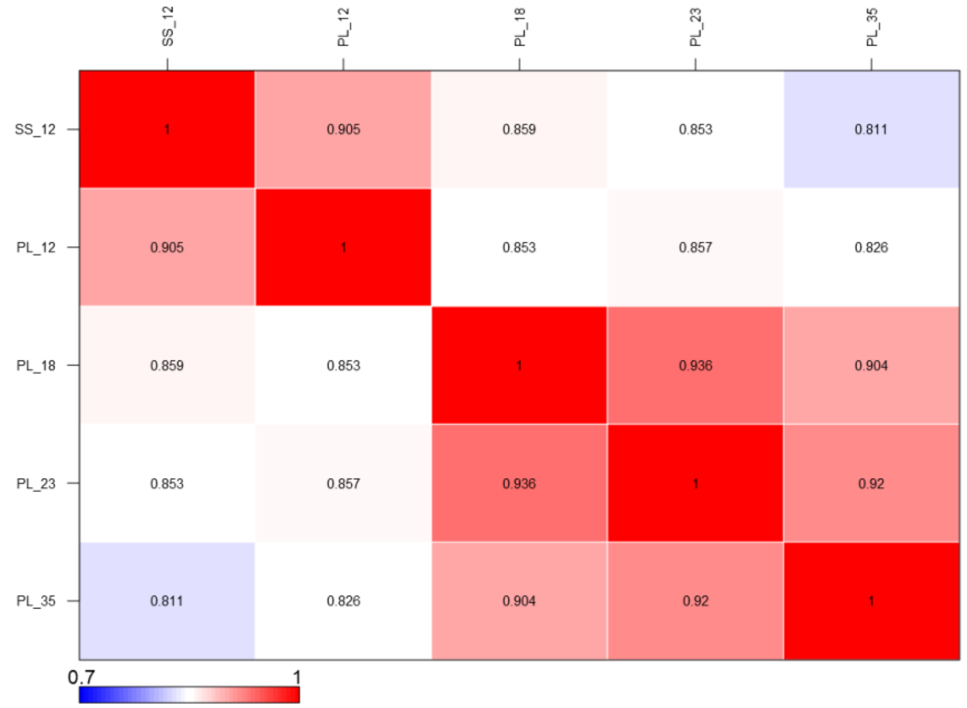

Fig.S4. Correlation matrix (shown as linear regression variances, R^2) between sessile (SS) and planktonic (PL) cell populations sampled simultaneously at 12 hours or when the PL fraction was sampled at 18, 23 or 36 hours. The proteome of SS and PL samples became increasingly divergent over time.

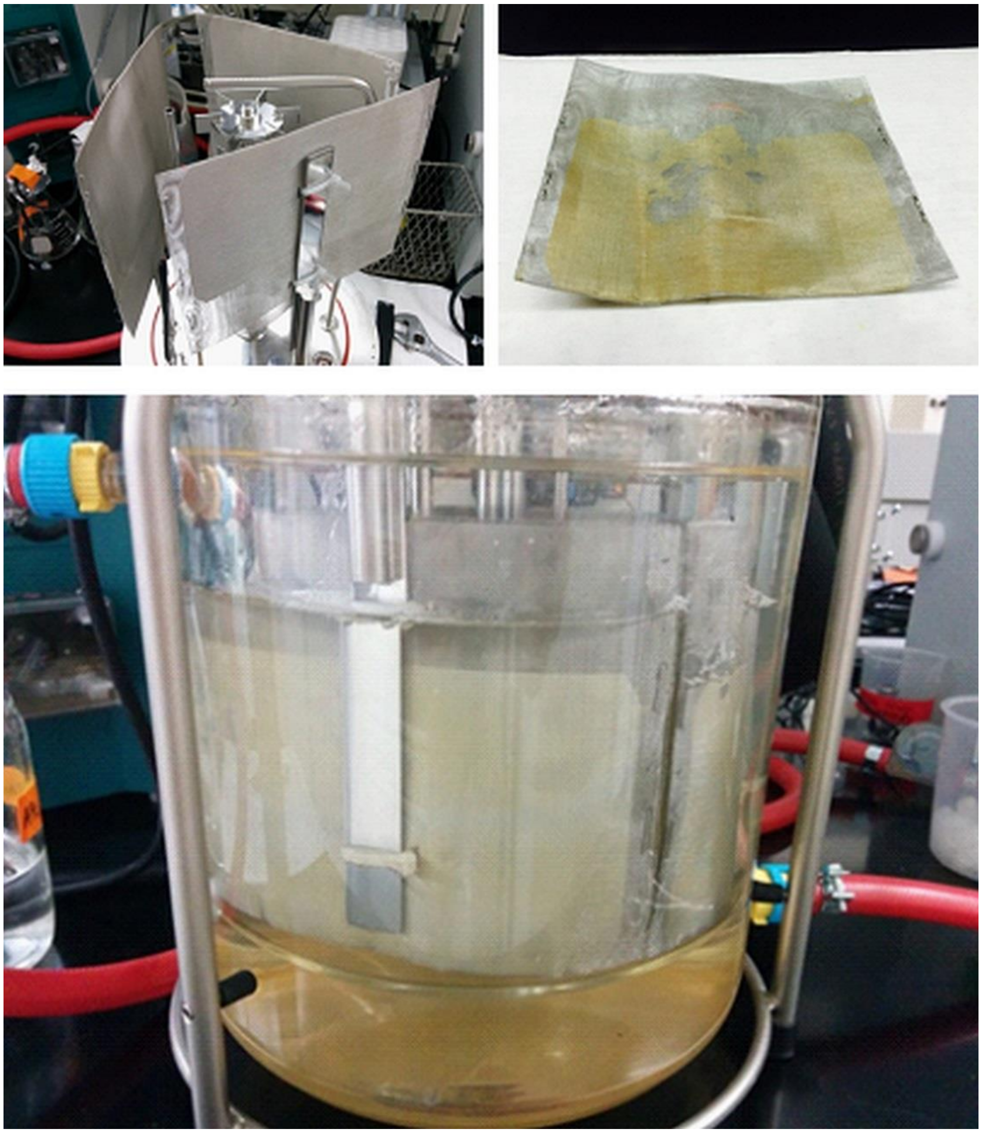

Fig. S5. Stainless steel mesh pockets with filter paper (biomass) sheets attached to bioreactor baffles (top left); biofilm develops on cellulose filter paper during fermentations in well-mixed liquid media (bottom); whole collection of biofilms and the cellulose support by extracting the stainless steel mesh pockets (top right).

Table S1. Summary statistics of library sequencing and mapping to reference genome GenBank CP000568.1

Library	Total number of reads	% Total unique filtered aligned reads	% Unique aligned on gene regions	% Unique aligned on intergenic regions
Sessile population 1	5614061	92.53%	75.86%	16.67%
Sessile population 2	5002547	94.35%	78.61%	15.74%
Sessile population 3	5272723	91.94%	75.31%	16.63%
Sessile population 4	5623221	93.00%	77.20%	15.80%
Planktonic population 1	6391262	90.92%	71.06%	19.86%
Planktonic population 2	5771770	90.99%	72.38%	18.61%
Planktonic population 3	6499808	92.39%	70.46%	21.93%
Planktonic population 4	5493563	92.39%	74.43%	17.96%

Table S2. Summary of descriptive statistics of (library-size non-normalized) gene read counts in the four replicate planktonic and sessile samples

Sample	Protein coding genes	Mean reads /gene	Std. Dev.of Mean	Std. Error of Mean	Sum of gene reads	Median	Maximum	Percentile		
								**90**	**95**	**99**
**PLANKTONIC**										
PL1	3299	667	3741	65	2201760	68	83941	805	1849	15028
PL2	3299	612	3608	63	2018090	59	89450	716	1653	12851
PL3	3299	673	3920	68	2219900	65	120187	838	2014	14684
PL4	3299	605	3754	65	1997170	58	118789	696	1678	11508
**Avg_PL**	**3299**	**639**	**3710**	**65**	**2109230**	**64**	**101205**	**779**	**1833**	**15229**
**SESSILE**										
SS1	3299	636	4468	78	2098860	118	161488	1170	2154	7145
SS2	3299	588	4226	74	1940830	102	156568	1009	1913	6336
SS3	3299	597	4289	75	1970350	111	168974	1066	1961	7004
SS4	3299	652	4591	80	2149800	118	178199	1163	2165	7909
**Avg_SS**	**3299**	**618**	**4384**	**76**	**2039960**	**114**	**166307**	**1110**	**2056**	**7094**

Table S3. Descriptive statistics of FPKM (fragments per kilobase per million gene reads) in the four replicate planktonic and sessile samples

Sample	Protein coding genes	Mean	Std. Dev.of Mean	Std. Error of Mean	Sum	Median	Maximum	Percentile		
								**90**	**95**	**99**
**PLANKTONIC**										
PL1	3299	330	1861	32	1088152	36	49005	398	961	6325
PL2	3299	331	1983	35	1091473	34	58312	380	945	6491
PL3	3299	337	1908	33	1111517	36	56439	401	1030	6506
PL4	3299	320	1817	32	1055656	36	50320	400	917	6080
**Avg_PL**	**3299**	**329**	**1874**	**33**	**1086699**	**36**	**53059**	**386.5**	**955**	**6614**
**SESSILE**										
SS1	3299	300	1141	20	990313.1	68	36220	648	1173	3369
SS2	3299	288	1111	19	951475.8	64	29947	581	1155	3262
SS3	3299	305	1187	21	1006630	68	32560	644	1126	3312
SS4	3299	300	1191	21	989751.4	67	32266	618	1109	3157
**Avg_SS**	**3299**	**298**	**1150**	**20**	**984543**	**67**	**32748**	**633**	**1134**	**3248**

Table S4. Genes that are significantly differential expressed between sessile and planktonic cell populations. Genes are grouped by metabolic pathway, cellular component and known or predicted functions based on reference genome annotation, association with ortholog groups in public databases KEGG and Biocyc, literature and experimental evidence. Log2(fold.difference) with positive or negative value for higher expression in sessile or planktonic populations, respectively. Raw gene reads are the number of sequencing fragments mapped to a gene region (sequencing paired ends are counted as one read); FPKM (fragments per kilobase per million reads) represents the relative gene expression in a population and was calculated as raw gene reads normalized by gene length (in Kb) and sequencing depth (i.e., total sequencing gene reads per sample). All data based on four biological replicates for each population.

Color code:

- (orange) Minimum two-fold higher expression in sessile (biofilm) cells
- (blue) Minimum two-fold higher expression in planktonic cells
- (light green) Significant differential expression with p<0.05
- (light pink) Top 5 percentile gene reads or expression (FPKM) within a population
- (magenta) Top 1 percentile gene reads or expression (FPKM) within a population

Locus tag	Syn.	Product (GenBank genome CP000568.1)	log2 (fold.diff.)	Signif. p	Raw gene reads PL	Raw gene reads SS	FPKM PL	FPKM SS
**Section 1.**								
**Carbohydrate metabolism (from cellodextrins to end-point metabolites)**								
**Intracellular cellodextrin hydrolysis and phosphorolytic cleavage**								
Cthe_2989		glycosyltransferase 36	2.36	1.6E-93	452	3712	73	615
Cthe_1265		phosphoglucomutase/phosphomannomutase alpha/beta/alpha domain I	1.44	4.1E-28	411	1777	112	501
Cthe_0040		glycoside hydrolase family 9	1.17	5.3E-16	158	560	28	103
Cthe_0071		glycoside hydrolase family 48	0.37	7.4E-02	147	302	25	53
Cthe_0212		beta-galactosidase	0.91	2.2E-08	462	1375	155	476
Cthe_2938		glucokinase, ROK family	1.57	1.7E-37	231	1096	115	563
Cthe_0275		glycosyltransferase 36	2.28	2.1E-50	648	4981	126	1003
**Glycolysis**								
Cthe_0217	pgi	Glucose-6-phosphate isomerase	2.27	1.2E-70	486	3726	171	1357
Cthe_0347		phosphofructokinase	3.46	2.6E-176	640	11308	244	4441
Cthe_1261		6-phosphofructokinase	0.89	5.3E-06	731	2144	354	1078
Cthe_2649		HpcH/HpaI aldolase	-1.95	2.8E-04	71	29	42	17
Cthe_0349		fructose-1,6-bisphosphate aldolase, class II	1.84	1.3E-62	1909	10879	976	5739
Cthe_0139	tpi	triosephosphate isomerase	2.28	3.3E-37	436	3417	275	2212
Cthe_0137		glyceraldehyde-3-phosphate dehydrogenase, type I	2.90	9.7E-86	821	9784	386	4745
Cthe_0138	pgk	Phosphoglycerate kinase	2.58	1.9E-100	827	7909	329	3248
Cthe_1435		Phosphoglycerate mutase	1.27	4.6E-05	14	55	12	46
Cthe_1292		proposed homoserine kinase	1.26	6.0E-23	280	1064	110	432
Cthe_0946		Phosphoglycerate mutase	1.40	1.1E-11	129	535	87	376
Cthe_0707		Phosphoglycerate mutase	-0.17	6.7E-01	163	231	124	180
Cthe_0140		phosphoglycerate mutase, 2,3-bisphosphoglycerate-independent	-3.70	7.3E-158	17447	2095	5378	670
Cthe_0143	eno	enolase	-0.60	5.4E-06	4568	4777	1667	1801
**Pyruvate fermentation**								
Cthe_2874		Phosphoenolpyruvate carboxykinase [GTP]	2.54	5.0E-35	1253	11643	328	3150
Cthe_0701		Conserved carboxylase region	-2.37	3.5E-70	8394	2555	2850	897
Cthe_0345		malate dehydrogenase	3.00	2.8E-153	392	4979	194	2549
Cthe_0344		malic protein NAD-binding	2.36	2.6E-74	936	7658	380	3202
Cthe_1308		pyruvate, phosphate dikinase	0.57	5.3E-04	2200	5182	393	958
Cthe_1053	ldh	L-lactate dehydrogenase	2.07	1.6E-16	35	238	18	122
Cthe_0505	pfl	formate acetyltransferase	-5.11	1.1E-70	67432	2902	14313	644
Cthe_2390	pfor	pyruvate/ketoisovalerate oxidoreductase, gamma subunit	3.74	2.4E-89	105	2251	85	1904
Cthe_2391		pyruvate ferredoxin/flavodoxin oxidoreductase, delta subunit	3.26	1.3E-103	100	1539	157	2477
Cthe_2392		pyruvate flavodoxin/ferredoxin oxidoreductase domain protein	3.01	4.1E-139	635	8165	254	3373
Cthe_0423	adh	iron-containing alcohol dehydrogenase	0.29	2.0E-01	5438	10436	979	1952
Cthe_1029		phosphate acetyltransferase	3.75	8.1E-50	38	827	17	375
Cthe_1028		Acetate kinase	3.83	1.0E-40	25	590	10	240
**Section 2.**								
**Hydrogen production**								
**Fe-Fe bifurcating hydrogenases**								
Cthe_0338	nuoE	NADH-quinone oxidoreductase, E subunit	2.24	2.8E-35	318	2419	304	2378
Cthe_0339		ATP-binding region ATPase domain protein	2.04	1.5E-42	281	1847	236	1596
Cthe_0340		ferredoxin	1.75	2.4E-22	168	907	217	1205
Cthe_0341	nuoF	Respiratory-chain NADH dehydrogenase domain 51 kDa subunit	1.91	2.2E-37	643	3840	170	1047

Locus tag	Syn.	Product (GenBank genome CP000568.1)	log2 (fold.diff.)	Signif. p	Raw gene reads PL	Raw gene reads SS	FPKM PL	FPKM SS
Cthe_0342	nuoG	hydrogenase, Fe-only	1.38	9.3E-19	786	3271	213	916
Cthe_0428	nuoE	NADH dehydrogenase (ubiquinone) 24 kDa subunit	1.16	2.1E-05	95	336	90	331
Cthe_0429	nuoF							
		Respiratory-chain NADH dehydrogenase domain 51 kDa subunit	0.99	1.4E-03	1953	6145	490	1606
Cthe_0430		hydrogenase, Fe-only	0.82	8.9E-03	1055	2935	292	846
**Fe-Fe hydrogenase**								
Cthe_3003		hydrogenase, Fe-only	1.35	3.8E-12	231	925	57	235
**Ech hydrogenase**								
Cthe_3020		NADH-ubiquinone oxidoreductase chain 49kDa	-0.58	1.2E-03	135	143	59	65
Cthe_3021		ech hydrogenase, subunit EchD, putative	0.85	3.6E-03	44	123	57	169
Cthe_3022		NADH ubiquinone oxidoreductase 20 kDa subunit	2.34	4.4E-09	6	48	6	53
Cthe_3023		respiratory-chain NADH dehydrogenase subunit 1	2.61	4.8E-20	13	126	7	70
Cthe_3024		NADH/Ubiquinone/plastoquinone (complex I)	2.89	3.1E-42	28	334	7	85
**Fe-Fe hydrogenase maturase**								
Cthe_0654	hydG	biotin and thiamin synthesis associated	-4.11	3.4E-110	3725	336	1276	119
Cthe_0042	hydF	small GTP-binding protein	2.90	1.7E-34	24	286	9	116
Cthe_1839	hydE	Radical SAM domain protein	-1.11	1.9E-21	784	574	350	265

Section 3.

ATP production by proton gradient

**V-type ATPase**								
Cthe_2262		V-type ATPase 116 kDa subunit	1.65	2.4E-22	254	1276	62	320
Cthe_2263		H+transporting two-sector ATPase C subunit	2.17	2.0E-17	27	196	28	205
Cthe_2264		V-type proton ATPase subunit E	2.15	1.2E-54	125	888	100	730
Cthe_2265		H+transporting two-sector ATPase C (AC39) subunit	2.29	3.5E-26	70	548	32	263
Cthe_2266		Vacuolar H+transporting two-sector ATPase F subunit	2.89	6.3E-13	6	76	9	116
Cthe_2267		V-type ATP synthase alpha chain	2.27	9.9E-41	95	728	25	202
Cthe_2268		V-type ATP synthase beta chain	1.99	1.5E-20	113	709	38	251
Cthe_2269		V-type ATP synthase subunit D	2.03	1.1E-29	70	452	49	331
**F-type ATPase**								
Cthe_2602		ATP synthase subunit a	1.40	3.4E-05	96	404	59	261
Cthe_2603		ATP synthase subunit c	2.55	6.3E-16	20	195	44	429
Cthe_2604		ATP synthase subunit b	2.16	6.3E-13	38	278	33	248
Cthe_2605		ATP synthase F1, delta subunit	2.09	1.5E-10	22	152	18	131
Cthe_2608		ATP synthase F1, beta subunit	1.84	1.2E-11	60	341	20	120
Cthe_2609		ATP synthase epsilon chain	1.71	2.2E-09	31	161	36	193

Section 4.

NAD (nicotinamide adenine dinucleotide) biosynthesis (via L-asparatate)

Cthe_2355		L-aspartate oxidase	2.69	3.1E-64	69	713	20	218
Cthe_2356		quinolinate synthetase complex, A subunit	2.18	2.5E-28	170	1233	88	659
Cthe_2354		nicotinate-nucleotide pyrophosphorylase	0.79	2.8E-03	99	276	57	162
Cthe_1241		nicotinate-nucleotide adenylyltransferase	0.82	1.4E-05	100	277	76	218
Cthe_0325		NAD+ synthetase	2.11	4.5E-36	86	586	21	149
**NAD to NADP**								
Cthe_0816		inorganic polyphosphate/ATP-NAD kinase	1.55	8.3E-13	90	422	49	238

Section 5.

ABC transporters

**Carbohydrate ABC transporters**								
Cthe_0391		ABC transporter related	-3.71	2.0E-83	17711	2122	5613	693
Cthe_0392		ABC-type transporter, integral membrane subunit	-3.60	5.2E-68	5575	716	2524	335
Cthe_0393	CbpA	periplasmic binding protein/LacI transcriptional regulator	-2.67	2.5E-40	10943	2690	5388	1370
Cthe_0394		iron-containing alcohol dehydrogenase	-2.81	1.0E-50	16859	3743	6837	1573
Cthe_0395		RbsD or FucU transport	-3.30	8.8E-43	7803	1221	8062	1312
Cthe_1018		ABC-type transporter, integral membrane subunit	1.48	1.1E-22	1100	4908	602	2765
Cthe_1019		ABC-type transporter, integral membrane subunit	1.73	5.8E-24	2268	12038	1098	5991
Cthe_1020	CbpB	extracellular solute-binding protein family 1	1.29	1.1E-16	23494	92277	8122	32748
Cthe_2125		ABC-type transporter, integral membrane subunit	0.01	1.0E+00	2	3	1	1
Cthe_2126		ABC-type transporter, integral membrane subunit	-0.99	2.0E-01	6	4	3	2
Cthe_2127		ATPase associated with various cellular activities AAA_3	-0.11	7.8E-01	43	63	25	39
Cthe_2128	CbpC	extracellular solute-binding protein family 1	-1.88	1.8E-11	59	25	20	9
Cthe_2446	CbpD	sugar ABC transporter substrate-binding protein	-2.43	8.4E-42	265	78	112	34
Cthe_2447		ABC transporter related	-2.41	5.0E-32	303	89	96	29

Locus tag	Syn.	Product (GenBank genome CP000568.1)	log2 (fold.diff.)	Signif. p	Raw gene reads PL	Raw gene reads SS	FPKM PL	FPKM SS
Cthe_2448		ABC-type transporter, integral membrane subunit	-2.68	2.7E-22	61	15	29	7
Cthe_2449		Phosphoglycerate mutase	-3.03	1.3E-13	60	11	46	9
Cthe_1576	Lbp	basic membrane lipoprotein	-1.35	1.0E-03	18	11	8	5
Cthe_1577		hypothetical protein	-0.09	8.8E-01	10	15	3	5
Cthe_1578		Radical SAM domain protein	-0.60	5.5E-01	2	2	1	1
Cthe_1579		ABC transporter related	-1.67	4.9E-02	4	2	1	1
Cthe_1580		ABC-type transporter, integral membrane subunit	-2.31	4.7E-02	2	1	1	0
Cthe_1581		ABC-type transporter, integral membrane subunit	-3.98	3.8E-03	3	0	1	0
**Maltose, oligosaccharide, polyol and lipid transport**								
Cthe_1862		ABC transporter related	2.29	4.6E-58	709	5503	301	2422
**Spermidine/Putrescine ABC transporters**								
Cthe_0747		extracellular solute-binding protein family 1	1.69	1.8E-08	77	400	34	184
Cthe_0748		ABC-type transporter, integral membrane subunit	1.58	2.1E-06	32	153	19	94
Cthe_0749		ABC-type transporter, integral membrane subunit	1.79	7.3E-08	41	229	23	135
Cthe_0750		spermidine/putrescine ABC transporter ATPase subunit	1.56	1.2E-06	64	306	29	141
Cthe_0751		Cupin 2 conserved barrel domain protein	2.29	1.1E-08	26	206	23	187
**Biotin ABC transporters**								
Cthe_0061		BioY protein	0.08	7.9E-01	105	176	90	157
Cthe_2934		ABC transporter related	1.95	2.1E-24	44	270	23	147
Cthe_2936		ABC transporter related	1.94	2.7E-20	35	211	19	122
Cthe_2937		ABC-type transporter, integral membrane subunit	1.60	1.1E-05	10	48	6	29
**Iron complex ABC transporters**								
Cthe_1752		ABC transporter related	-0.64	1.7E-03	104	105	61	64
Cthe_1753		ABC-type transporter, integral membrane subunit	-0.15	5.7E-01	37	52	17	25
Cthe_1754		ABC-type transporter, periplasmic subunit	1.06	4.5E-06	28	91	14	47
**Zinc ABC transporters**								
Cthe_0547		ABC-type metal ion transporter, periplasmic subunit	0.29	1.4E-01	169	327	84	168
Cthe_0548		ABC transporter related	-0.12	5.8E-01	115	166	69	105
Cthe_0549		ABC-type transporter, integral membrane subunit	-0.12	6.4E-01	74	108	41	62
**Nickel and cobalt ABC transporters**								
Cthe_1801		ABC transporter related	-0.06	8.7E-01	23	36	14	23
Cthe_1802		cobalt ABC transporter, inner membrane subunit CbiQ	-0.07	8.0E-01	48	72	28	44
Cthe_1803		cobalamin (vitamin B12) biosynthesis CbiM protein	0.29	2.2E-01	104	203	47	94
**Na+ ABC transporters**								
Cthe_2942		ABC transporter related	0.90	4.7E-05	36	105	22	68
Cthe_2943		ABC-2 type transporter	0.55	1.5E-02	58	135	23	54
**Sulfate ABC transporters**								
Cthe_2531		sulfate ABC transporter, periplasmic sulfate-binding protein	-1.08	7.0E-03	32	24	14	11
Cthe_2532		sulfate ABC transporter, inner membrane subunit CysT	-2.74	2.4E-19	90	21	50	12
Cthe_2533		sulfate ABC transporter, inner membrane subunit CysW	0.11	9.1E-01	2	3	1	2
Cthe_2534		sulfate ABC transporter, ATPase subunit	-1.09	1.8E-02	13	9	6	4
**Iron (III) ABC transporters**								
Cthe_1586		ABC-type transporter, integral membrane subunit	-1.28	3.6E-03	16	10	4	3
Cthe_1587		ABC transporter related	-1.91	1.3E-04	13	5	6	2
Cthe_1588		extracellular solute-binding protein family 1	-1.29	5.9E-04	33	22	14	9
**Phosphate uptake regulon and phosphate ABC transporter**								
Cthe_1599		PAS sensor protein	1.17	1.7E-09	52	185	15	54
Cthe_1600		response regulator receiver	0.98	1.7E-08	109	342	77	249
Cthe_1601		phosphate transport system regulatory protein PhoU	1.63	2.8E-11	65	317	47	239
Cthe_1602		phosphate ABC transporter, ATPase subunit	0.12	7.8E-01	21	37	13	24
Cthe_1603		phosphate ABC transporter, inner membrane subunit PstA	0.03	9.6E-01	6	10	3	6
Cthe_1604		phosphate ABC transporter, inner membrane subunit PstC	1.63	8.1E-02	1	6	1	3
Cthe_1605		ABC-type phosphate transport system periplasmic component-like protein	0.80	7.6E-02	8	21	4	12

Locus tag	Syn.	Product (GenBank genome CP000568.1)	log2 (fold.diff.)	Signif. p	Raw gene reads PL	Raw gene reads SS	FPKM PL	FPKM SS
**Urea ABC transporters**								
Cthe_1819		urea ABC transporter, ATP-binding protein UrtE	-1.23	4.1E-02	8	5	5	4
Cthe_1820		urea ABC transporter, ATP-binding protein UrtD	-1.44	2.4E-02	7	4	4	2
Cthe_1821		urea ABC transporter, permease protein UrtC	-1.03	1.8E-02	16	12	7	5
Cthe_1822		urea ABC transporter, permease protein UrtB	-0.56	4.1E-01	6	6	3	3
Cthe_1823		urea ABC transporter, urea binding protein	-0.74	2.2E-01	7	6	2	2
**Oligopeptide ABC transporters**								
Cthe_2961		ABC-type transporter, periplasmic subunit	-1.19	8.3E-04	62	43	18	13
Cthe_2962		oligopeptide/dipeptide ABC transporter, ATPase subunit	0.18	7.2E-01	20	35	9	17
Cthe_2963		oligopeptide/dipeptide ABC transporter, ATPase subunit	-0.14	7.3E-01	29	42	13	20
Cthe_2964		ABC-type transporter, integral membrane subunit	-0.24	5.1E-01	45	60	20	27
Cthe_2965		ABC-type transporter, integral membrane subunit	0.36	2.5E-01	55	111	28	59
**Alkanesulfonate ABC transporters**								
Cthe_1571		ABC-type transporter, integral membrane subunit	-0.70	1.0E-02	88	85	39	39
Cthe_1572		ABC transporter related	-0.55	7.7E-02	34	36	20	22
**Amino acid ABC transporters**								
Cthe_1456		ABC transporter related	2.13	5.4E-11	18	127	11	79
Cthe_1457		polar amino acid ABC transporter, inner membrane subunit	2.45	3.1E-29	32	277	23	205
Cthe_1458		ABC-type transporter, periplasmic subunit family 3	1.94	6.2E-24	176	1088	104	658
Cthe_2278		ABC-type transporter, periplasmic subunit family 3	1.06	5.7E-10	101	334	55	189
Cthe_2279		polar amino acid ABC transporter, inner membrane subunit	1.11	3.3E-05	23	78	15	54
Cthe_2280		ABC transporter related	2.14	1.0E-08	7	52	4	31
**Nitrate/Sulfonate/Bicarbonate ABC transporters**								
Cthe_2116		ABC-type transporter, integral membrane subunit	-0.36	2.3E-01	37	46	23	29
Cthe_2117		ABC transporter, substrate-binding protein, putative	-0.41	2.2E-01	37	44	17	21
Cthe_2118		ABC transporter related	-1.05	3.7E-04	43	33	27	21
Cthe_2802		ABC-type transporter, periplasmic subunit family 3	-2.38	8.9E-17	123	36	59	18
Cthe_2803		ABC-type transporter, integral membrane subunit	-2.31	4.5E-11	46	14	28	9
Cthe_2804		ABC transporter related	-2.88	6.3E-20	237	49	144	31
**Other ABC transporter proteins**								
Cthe_0384		ABC transporter related	-2.03	5.3E-02	3	1	2	1
Cthe_0396		ABC transporter related	0.96	1.5E-04	31	97	9	28
Cthe_0397		ABC transporter related	1.89	3.6E-14	19	112	5	31
Cthe_0534		ABC-type bacteriocin transporter	-2.33	3.6E-28	204	64	44	14
Cthe_0539		ABC transporter related	-0.02	9.6E-01	16	24	11	17
Cthe_0819		ABC transporter related	-0.34	1.1E-01	105	132	57	74
Cthe_0910		ABC-type transporter, periplasmic subunit	0.26	1.8E-01	166	317	47	93
Cthe_1189		ABC transporter related	2.57	2.3E-14	9	90	5	45
Cthe_1415		ABC transporter related	-1.09	5.9E-03	20	15	11	8
Cthe_1500		ABC transporter related	1.08	1.2E-09	74	250	19	65
Cthe_1501		ABC transporter transmembrane region	0.40	7.8E-02	65	136	18	38
Cthe_1526		ABC transporter related	0.32	5.6E-01	6	12	4	8
Cthe_1536		ABC transporter related	-1.63	1.1E-06	32	16	17	9
Cthe_1555		NLPA lipoprotein	0.05	8.6E-01	1636	2696	881	1495
Cthe_1556		ABC-type transporter, integral membrane subunit	0.06	8.4E-01	1588	2606	1061	1803
Cthe_1557		ABC transporter related	0.37	3.2E-02	653	1333	376	795
Cthe_1563		ABC transporter related	0.05	9.1E-01	19	31	13	22
Cthe_1570		ABC-type transporter, periplasmic subunit family 3	1.04	1.6E-03	59	195	26	89
Cthe_1667		ABC-2 type transporter	0.54	7.4E-01	0	1	0	1
Cthe_1668		ABC transporter related	-1.08	4.3E-01	1	1	1	0
Cthe_1685		ABC transporter related	-3.34	8.5E-24	405	60	111	17
Cthe_1763		ABC transporter related	0.60	5.3E-04	155	374	99	247
Cthe_1799		ABC transporter related	2.75	8.8E-60	62	670	15	171
Cthe_2110		ABC transporter related	-0.39	5.5E-01	6	7	4	5
Cthe_2270		ABC transporter related	0.41	2.1E-01	19	39	5	12
Cthe_2290		ABC transporter related	-2.11	3.2E-02	4	1	2	1
Cthe_2573		ABC transporter related	-1.53	3.6E-03	19	10	12	6
Cthe_2574		ABC-type transporter, integral membrane subunit	-3.09	3.6E-03	4	1	2	0
Cthe_2664		ABC-1 domain-containing protein	-0.41	3.7E-02	179	214	50	61

Locus tag	Syn.	Product (GenBank genome CP000568.1)	log2 (fold.diff.)	Signif. p	Raw gene reads PL	Raw gene reads SS	FPKM PL	FPKM SS
Cthe_2706		ABC transporter related	1.76	5.3E-45	184	993	79	438
Cthe_2707		ABC-2 type transporter	1.68	8.9E-15	36	184	24	125
Cthe_2708		ABC-type uncharacterized transport system	1.47	1.6E-27	252	1104	84	380
Cthe_2789		ABC-type transporter, periplasmic subunit family 3	-1.41	2.5E-02	8	5	4	2
Cthe_2790		ABC transporter related	-0.48	5.9E-01	3	3	1	2
Cthe_2791		ABC-type transporter, integral membrane subunit	-0.99	3.0E-01	3	2	1	1
Cthe_2970		ABC transporter related	0.38	8.6E-02	47	96	26	55
Cthe_2982		ABC-type transporter, periplasmic subunit family 3	-1.06	4.6E-01	0	0	0	0
Cthe_2998		ABC transporter related	2.01	8.1E-36	69	442	19	125
Cthe_3066		ABC transporter related	-1.31	8.3E-16	290	186	190	126
Cthe_3147		ABC transporter related	2.46	4.0E-16	17	155	5	43
Cthe_3148		ABC transporter related	2.15	5.5E-16	21	148	5	38
Cthe_3170		ABC transporter related	0.82	1.6E-01	4	12	3	9
**Section 6.**								
**Amino acid biosynthesis**								
**L-arginine (via L-glutamate, L-glutamine and L-ornithine)**								
Cthe_0081		Arginine biosynthesis bifunctional protein ArgJ	2.57	2.9E-46	82	783	32	318
Cthe_0949		carbamoyl-phosphate synthase, large subunit	1.98	2.7E-16	277	1722	40	263
Cthe_0950		carbamoyl-phosphate synthase, small subunit	2.40	9.0E-20	66	552	29	253
Cthe_0178		Argininosuccinate lyase	2.79	9.1E-32	132	1469	45	521
Cthe_0179		Argininosuccinate synthase	3.28	2.4E-42	122	1917	47	768
Cthe_1863		N-acetyl-gamma-glutamyl-phosphate reductase	5.41	2.8E-78	21	1461	9	686
Cthe_1864		acetylglutamate kinase	4.85	9.2E-43	18	867	9	462
Cthe_1866		acetylornithine and succinylornithine aminotransferase	3.82	4.5E-46	98	2262	39	918
Cthe_1867		carbamoyl-phosphate synthase, small subunit	3.45	5.1E-44	112	1972	50	906
Cthe_1868		carbamoyl-phosphate synthase, large subunit	3.43	1.5E-42	258	4477	38	683
Cthe_1869		Ornithine carbamoyltransferase	2.30	3.0E-24	294	2323	151	1231
Cthe_3363		hypothetical protein	0.49	4.3E-01	5	11	11	25
**L-asparate (via oxaloacetate)**								
Cthe_0580		aminotransferase class I and II	1.39	3.9E-16	74	307	29	126
Cthe_0755		aminotransferase class I and II	2.15	5.8E-11	333	2394	134	991
**L-asparagine (via L-aspartate)**								
Cthe_0069		Aspartate--ammonia ligase	2.15	4.4E-53	114	800	53	383
Cthe_0556		asparagine synthase (glutamine-hydrolyzing)	-1.27	3.7E-02	11	7	3	2
**L-lysine (via L-aspartate)**								
Cthe_1375		aspartate kinase	1.78	9.5E-24	130	713	46	258
Cthe_0961		aspartate-semialdehyde dehydrogenase	2.77	3.7E-93	182	1972	86	969
Cthe_0962		dihydrodipicolinate synthase	1.99	1.2E-27	99	629	53	351
Cthe_0922		diaminopimelate dehydrogenase	1.29	1.3E-13	92	359	44	175
Cthe_0683		diaminopimelate decarboxylase	1.55	9.2E-08	102	478	37	179
**Phenylalanine (via chorismate)**								
Cthe_2260		prephenate dehydratase	2.31	2.7E-30	33	260	19	152
Cthe_0580		aminotransferase class I and II	1.39	3.9E-16	74	307	29	126
Cthe_0755		aminotransferase class I and II	2.15	5.8E-11	333	2394	134	991
**L-tyrosine (via chorismate)**								
Cthe_0711		chorismate mutase	0.99	2.1E-06	234	744	312	1022
Cthe_1796		Prephenate dehydrogenase	1.98	3.9E-49	352	2215	152	988
**L-histidine (via phosphoribosyl pyrophosphate, PRPP)**								
Cthe_2880		ATP phosphoribosyltransferase regulatory subunit	1.24	5.1E-07	98	366	37	143
Cthe_2881		ATP phosphoribosyltransferase	0.81	1.8E-03	68	188	50	143
Cthe_2889		Histidine biosynthesis bifunctional protein hisIE	1.04	1.7E-09	79	256	56	190
Cthe_2888		Imidazole glycerol phosphate synthase subunit hisF	1.38	7.4E-13	74	306	46	197
Cthe_2887		Phosphoribosylformimino-5-aminoimidazole carboxamide ribotide isomerase	1.54	3.1E-14	114	527	75	362
Cthe_2886		Imidazole glycerol phosphate synthase subunit hisH	1.77	1.9E-16	39	214	30	170
Cthe_2884		Imidazoleglycerol-phosphate dehydratase	1.46	4.2E-14	53	233	43	195
Cthe_2883		histidinol-phosphate aminotransferase	0.27	2.4E-01	135	256	59	116
Cthe_0610		Histidinol-phosphate aminotransferase	0.57	7.8E-02	19	45	8	21
Cthe_0724		histidinol phosphate phosphatase HisJ family	0.10	8.1E-01	67	114	40	70
Cthe_2882		histidinol dehydrogenase	-0.01	9.8E-01	194	304	70	114

Locus tag	Syn.	Product (GenBank genome CP000568.1)	log2 (fold.diff.)	Signif. p	Raw gene reads PL	Raw gene reads SS	FPKM PL	FPKM SS
**L-valine and L-leucine (via pyruvate)**								
Cthe_2516		acetolactate synthase, large subunit, biosynthetic type	1.17	1.1E-16	323	1147	94	345
Cthe_2517		acetolactate synthase, small subunit	1.68	5.2E-23	96	485	88	465
Cthe_2714		acetolactate synthase, large subunit, biosynthetic type	0.74	3.8E-04	736	1926	208	567
Cthe_2518		ketol-acid reductoisomerase	2.24	1.3E-63	590	4411	282	2177
Cthe_2713		dihydroxy-acid dehydratase	0.29	2.6E-01	531	1019	151	300
Cthe_1391		2-isopropylmalate synthase	-1.73	6.5E-08	5519	2531	1709	817
Cthe_2519		2-isopropylmalate synthase/homocitrate synthase family protein	1.91	2.7E-35	231	1375	67	416
Cthe_2210		3-isopropylmalate dehydratase, small subunit	-0.57	5.4E-03	352	374	293	322
Cthe_2211		3-isopropylmalate dehydratase large subunit	-0.61	6.6E-03	1130	1157	425	452
Cthe_2209		3-isopropylmalate dehydrogenase	-0.06	7.9E-01	438	659	190	297
Cthe_0856		branched-chain amino acid aminotransferase	2.60	1.1E-58	200	1936	88	885
**L-isoleucine and L-threonine (via L-asparate and L-homoserine)**								
Cthe_1375		aspartate kinase	1.78	9.5E-24	130	713	46	258
Cthe_0961		aspartate-semialdehyde dehydrogenase	2.77	3.7E-93	182	1972	86	969
Cthe_0290		homoserine dehydrogenase	-1.76	2.5E-15	969	451	355	171
Cthe_1376		homoserine dehydrogenase	2.88	3.9E-57	50	592	19	232
Cthe_1381		threonine synthase	0.31	1.1E-01	802	1566	252	511
Cthe_2516		acetolactate synthase, large subunit, biosynthetic type	1.17	1.1E-16	323	1147	94	345
Cthe_2517		acetolactate synthase, small subunit	1.68	5.2E-23	96	485	88	465
Cthe_2714		acetolactate synthase, large subunit, biosynthetic type	0.74	3.8E-04	736	1926	208	567
Cthe_2518		ketol-acid reductoisomerase	2.24	1.3E-63	590	4411	282	2177
Cthe_2713		dihydroxy-acid dehydratase	0.29	2.6E-01	531	1019	151	300
Cthe_0856		branched-chain amino acid aminotransferase	2.60	1.1E-58	200	1936	88	885
**L-serine (via 3-phosphoglycerate)**								
Cthe_3035		D-isomer specific 2-hydroxyacid dehydrogenase NAD-binding	1.05	1.1E-08	758	2507	308	1047
Cthe_0295		phosphoserine aminotransferase	1.24	2.5E-07	395	1507	168	658
**Glycine (via L-serine)**								
Cthe_1058		glycine hydroxymethyltransferase	2.77	1.1E-68	384	4145	146	1642
**L-glutamate (via 2-oxoglutarate and ammonium)**								
Cthe_0374		Glu/Leu/Phe/Val dehydrogenase	2.42	6.8E-18	383	3263	136	1203
**L-glutamate (via 2-oxoglutarate and L-glutamine)**								
Cthe_0201		glutamate synthase alpha subunit domain protein	-4.79	4.6E-53	1375	73	896	50
Cthe_0198		ferredoxin-dependent glutamate synthase	-1.30	1.4E-03	29	18	9	6
Cthe_0197		glutamine amidotransferase class-II	-0.18	7.6E-01	7	10	3	5
**L-glutamine (via L-glutamate)**								
Cthe_0202		glutamine synthetase, type I	-3.79	1.4E-69	1007	113	360	42
Cthe_0196		glutamine synthetase catalytic region	0.99	2.4E-04	39	125	9	29
Cthe_0863		glutamine synthetase catalytic region	1.76	5.4E-18	107	581	24	134
Cthe_1539		glutamine synthetase catalytic region	-0.67	2.9E-01	7	7	2	2
**L-cysteine (via L-serine)**								
Cthe_2066		serine O-acetyltransferase	-3.96	1.6E-81	2395	236	1511	156
Cthe_1840		cysteine synthase A	-0.13	3.4E-01	899	1292	454	679
**L-alanine (via L-cysteine)**								
Cthe_0655		cysteine desulfurase family protein	-2.07	2.1E-32	1498	564	620	240
Cthe_0265		aminotransferase class V	-0.79	8.7E-03	626	576	266	251
**Methionine (via L-homoserine and L-cysteine)**								
Cthe_1845		homoserine O-succinyltransferase	-1.09	1.3E-20	1850	1368	955	732
Cthe_2799		Cys/Met metabolism pyridoxal-phosphate-dependent protein	-1.68	2.4E-16	1108	538	452	229
Cthe_0645		homocysteine S-methyltransferase	-0.62	5.0E-03	760	782	149	159
**L-proline (via L-glutamate)**								
Cthe_1766		glutamate 5-kinase	1.91	1.2E-24	78	470	44	276
Cthe_0262		Gamma-glutamyl phosphate reductase	-0.52	9.5E-04	1390	1535	510	582
Cthe_0672		pyrroline-5-carboxylate reductase	-0.84	4.4E-04	422	374	246	225

Locus tag	Syn.	Product (GenBank genome CP000568.1)	log2 (fold.diff.)	Signif. p	Raw gene reads PL	Raw gene reads SS	FPKM PL	FPKM SS
**L-tryptophan (via indole and L-serine)**								
Cthe_1411		Tryptophan synthase alpha chain	-1.16	8.6E-06	723	513	449	326
Cthe_1412		Tryptophan synthase beta chain	-1.56	1.5E-10	895	472	359	196
Cthe_1211		Tryptophan synthase beta chain	1.55	1.0E-27	1332	6211	464	2229

Section 7.

Ribosomal proteins

Locus tag	Syn.	Product (GenBank genome CP000568.1)	log2 (fold.diff.)	Signif. p	Raw gene reads PL	Raw gene reads SS	FPKM PL	FPKM SS
Cthe_2902		30S ribosomal protein S10	3.42	2.4E-103	88	1494	133	2353
Cthe_2903		50S ribosomal protein L3	3.13	5.1E-73	138	1920	102	1473
Cthe_2904		ribosomal protein L4/L1e	3.21	5.7E-49	120	1769	90	1385
Cthe_2905		Ribosomal protein L25/L23	2.39	3.3E-41	88	730	118	1014
Cthe_2906		ribosomal protein L2	2.62	3.5E-49	247	2387	141	1417
Cthe_2907		ribosomal protein S19	2.64	7.3E-33	64	630	105	1087
Cthe_2908		ribosomal protein L22	2.31	1.5E-37	120	946	151	1228
Cthe_2909		ribosomal protein S3	2.40	7.0E-51	294	2472	206	1784
Cthe_2910		50S ribosomal protein L16	2.49	8.8E-47	128	1136	139	1276
Cthe_2911		ribosomal protein L29	2.34	5.3E-24	34	271	78	652
Cthe_2912		30S ribosomal protein S17	2.26	2.8E-38	109	832	202	1588
Cthe_2913		ribosomal protein L14	2.27	1.2E-29	79	610	102	813
Cthe_2914		ribosomal protein L24	2.20	7.1E-58	176	1276	237	1786
Cthe_2915		ribosomal protein L5	2.66	1.2E-54	267	2688	232	2407
Cthe_2916		ribosomal protein S14	2.36	3.7E-19	22	177	55	467
Cthe_2917		ribosomal protein S8	2.54	6.7E-58	158	1458	187	1794
Cthe_2918		ribosomal protein L6	2.34	6.4E-30	263	2130	226	1891
Cthe_2919		ribosomal protein L18	2.13	3.0E-23	187	1305	241	1739
Cthe_2920		ribosomal protein S5	2.22	1.8E-32	168	1246	159	1220
Cthe_2921		ribosomal protein L30	2.45	1.5E-29	51	440	133	1205
Cthe_2922		ribosomal protein L15	2.43	8.9E-33	121	1042	130	1158
Cthe_2928		ribosomal protein L36	2.00	2.6E-35	59	373	246	1617
Cthe_2929		30S ribosomal protein S13	2.24	1.5E-63	307	2294	392	3031
Cthe_2930		30S ribosomal protein S11	2.58	3.7E-81	148	1401	173	1702
Cthe_2931		ribosomal protein S4	2.36	6.1E-65	462	3746	349	2935
Cthe_2933		ribosomal protein L17	2.08	2.9E-41	476	3211	431	2993
Cthe_1782		ribosomal protein S9	2.24	4.5E-38	273	2064	331	2584
Cthe_1783		ribosomal protein L13	3.07	6.8E-75	104	1381	113	1555
Cthe_2726		ribosomal protein L7Ae/L30e/S12e/Gadd45	2.84	5.1E-44	27	305	53	624
Cthe_2727		ribosomal protein S12	3.23	1.3E-108	124	1847	137	2117
Cthe_2728		ribosomal protein S7	3.23	1.2E-113	407	6075	410	6342
Cthe_2720		ribosomal protein L11	1.91	8.5E-33	332	1992	371	2294
Cthe_2721		ribosomal protein L1	2.46	2.9E-55	279	2460	191	1730
Cthe_2722		50S ribosomal protein L10	2.79	4.8E-90	298	3268	264	2992
Cthe_2723		ribosomal protein L7/L12	2.51	1.8E-102	291	2629	355	3319
Cthe_1006		ribosomal protein S2	2.58	2.2E-86	455	4295	283	2776
Cthe_0417		ribosomal protein S15	2.24	1.2E-25	36	272	65	507
Cthe_1223		50S ribosomal protein L20	1.76	1.1E-27	212	1135	282	1573
Cthe_1224		50S ribosomal protein L35	2.65	1.7E-30	37	371	88	918
Cthe_2370		50S ribosomal protein L34	2.27	4.9E-27	84	650	294	2366
Cthe_2172		50S ribosomal protein L31	2.45	2.9E-14	52	461	115	1060
Cthe_1026		ribosomal protein L32	2.22	3.2E-22	544	4126	1301	10109
Cthe_2257		ribosomal protein L9	1.05	8.7E-10	80	263	85	288
Cthe_2185		30S ribosomal protein S18	2.78	6.8E-35	34	376	57	648
Cthe_2187		30S ribosomal protein S6	3.20	8.5E-97	63	929	105	1584
Cthe_1279		50S ribosomal protein L28	2.92	1.3E-51	372	4494	933	11686
Cthe_0160		50S ribosomal protein L21	0.53	2.8E-03	690	1596	1059	2513
Cthe_0162		50S ribosomal protein L27	1.29	7.7E-19	551	2151	939	3785
Cthe_0769		ribosomal protein S16	2.25	2.1E-26	74	560	142	1120
Cthe_0765		ribosomal protein L19	1.06	4.9E-08	865	2879	1198	4110
Cthe_1039		ribosomal protein S20	3.87	2.2E-78	257	6053	353	8574
Cthe_1348		30S ribosomal protein S21	-0.03	9.4E-01	3069	4780	8291	13327
Cthe_1298		Ribosomal protein L25	-1.91	8.2E-05	17	7	25	10
Cthe_0992		ribosomal protein L7Ae/L30e/S12e/Gadd45	-0.58	1.1E-03	139	147	186	203
**Ribosomal protein processing**								
Cthe_0942		Ribosomal protein S12 methylthiotransferase rimO	0.90	1.1E-06	354	1045	123	376
Cthe_1320		Ribosomal protein L11 methyltransferase	1.13	4.9E-06	53	184	27	96
Cthe_1774		ribosomal-protein-alanine acetyltransferase	1.03	3.8E-06	40	129	41	137
**Ribosomal RNA processing**								
Cthe_0572		Ribosomal RNA large subunit methyltransferase N	0.23	1.5E-01	325	603	147	282

Locus tag	Syn.	Product (GenBank genome CP000568.1)	log2 (fold.diff.)	Signif. p	Raw gene reads PL	Raw gene reads SS	FPKM PL	FPKM SS
Cthe_1319		Ribosomal RNA small subunit methyltransferase E	0.43	6.7E-02	87	187	55	123
Cthe_2092		Ribosomal RNA small subunit methyltransferase A	1.29	2.2E-05	21	82	11	46
Cthe_2101		Ribosomal RNA small subunit methyltransferase I	0.69	4.8E-04	97	249	55	145
Cthe_2363		Ribosomal RNA small subunit methyltransferase G	1.61	2.9E-11	33	161	21	108
Cthe_3180		ribosomal RNA methyltransferase	1.87	1.9E-16	52	302	27	162
Cthe_0767		Ribosome maturation factor rimM	1.70	2.0E-05	28	151	26	141

Section 8.

Aminoacyl-tRNA charging

Locus tag	Syn.	Product (GenBank genome CP000568.1)	log2 (fold.diff.)	Signif. p	Raw gene reads PL	Raw gene reads SS	FPKM PL	FPKM SS
Cthe_0070		Asparaginyl-tRNA synthetase	1.48	1.8E-25	268	1176	91	413
Cthe_0214		Phenylalanyl-tRNA synthetase alpha chain	1.71	1.9E-16	70	367	33	176
Cthe_0215		Phenylalanyl-tRNA synthetase beta chain	1.69	3.1E-32	208	1066	41	218
Cthe_0324		valyl-tRNA synthetase	2.86	9.1E-45	98	1132	18	209
Cthe_0648		glutamyl-tRNA synthetase	2.01	1.3E-41	156	1000	45	299
Cthe_0686		tryptophanyl-tRNA synthetase	1.64	3.3E-17	129	644	62	318
Cthe_0723		Tyrosyl-tRNA synthetase	0.14	5.1E-01	388	680	150	271
Cthe_0787		Isoleucyl-tRNA synthetase	2.19	7.8E-47	269	1948	46	342
Cthe_0917		Glutaminyl-tRNA synthetase	2.14	5.0E-52	379	2650	103	746
Cthe_1228		threonyl-tRNA synthetase	1.89	4.1E-28	392	2327	98	596
Cthe_1237		leucyl-tRNA synthetase	1.54	7.0E-47	832	3857	159	762
Cthe_1312		glycyl-tRNA synthetase	1.43	4.7E-26	472	2020	161	712
Cthe_1331		aspartyl-tRNA synthetase	1.85	1.4E-36	249	1431	66	392
Cthe_1332		Histidyl-tRNA synthetase	1.43	9.9E-24	149	641	56	250
Cthe_1543		aspartyl-tRNA synthetase	1.11	3.2E-03	10	36	3	10
Cthe_1935		arginyl-tRNA synthetase	-0.60	1.6E-03	843	877	234	254
Cthe_2065		cysteinyl-tRNA synthetase	-3.75	5.4E-137	4473	522	1506	182
Cthe_2096		methionyl-tRNA synthetase	2.56	1.6E-93	144	1355	35	338
Cthe_2381		Seryl-tRNA synthetase	0.68	1.4E-10	511	1298	190	500
Cthe_2815		lysyl-tRNA synthetase	2.65	1.0E-88	262	2620	78	801
Cthe_2947		prolyl-tRNA synthetase	2.19	1.1E-46	140	1023	39	291
Cthe_3200		alanyl-tRNA synthetase	1.78	4.8E-35	330	1798	59	333

Section 9.

Translation initiation and elongation factors

Locus tag	Syn.	Product (GenBank genome CP000568.1)	log2 (fold.diff.)	Signif. p	Raw gene reads PL	Raw gene reads SS	FPKM PL	FPKM SS
Cthe_2927		Translation initiation factor IF-1	2.32	2.4E-38	45	359	98	809
Cthe_0991		translation initiation factor IF-2	-0.23	1.7E-01	1503	2027	229	319
Cthe_1225		Translation initiation factor IF-3	2.13	9.9E-32	241	1699	233	1685
Cthe_0847		translation elongation factor P	1.74	1.3E-33	619	3284	524	2882
Cthe_1005		translation elongation factor Ts	2.37	1.0E-85	302	2499	222	1890
Cthe_1794		translation elongation factor G	-1.64	4.9E-24	2143	1079	487	254
Cthe_2729		translation elongation factor G	2.90	7.1E-100	764	9047	173	2116
Cthe_2730		translation elongation factor Tu	2.19	5.6E-46	3229	23722	1282	9680
**SsrA quality control (fidelity of protein synthesis)**								
Cthe_2748	SmpB	SsrA-binding protein	1.56	5.3E-22	100	470	102	497

Section 10.

Chaperones-like function for the protection of newly synthesised polypeptide chains

Locus tag	Syn.	Product (GenBank genome CP000568.1)	log2 (fold.diff.)	Signif. p	Raw gene reads PL	Raw gene reads SS	FPKM PL	FPKM SS
Cthe_1321		Chaperone protein dnaJ	1.70	1.2E-17	115	594	47	251
Cthe_1322		Chaperone protein dnaK	0.89	2.2E-09	1124	3274	291	882
Cthe_1323		GrpE protein	0.98	1.6E-04	256	797	177	577
Cthe_1852		33 kDa chaperonin	1.73	1.1E-20	118	629	63	345
Cthe_2891		10 kDa chaperonin	2.61	6.0E-33	117	1130	193	1959
Cthe_2892		60 kDa chaperonin	2.27	1.5E-43	769	5882	224	1781
Cthe_0068		peptidyl-prolyl cis-trans isomerase cyclophilin type	2.04	3.0E-22	75	495	58	396
Cthe_2739		Trigger factor	-1.97	3.1E-08	5055	1995	1848	757

Section 11.

Protein secretion (Sec system)

Locus tag	Syn.	Product (GenBank genome CP000568.1)	log2 (fold.diff.)	Signif. p	Raw gene reads PL	Raw gene reads SS	FPKM PL	FPKM SS
**Sec system**								
Cthe_0926	FtsY	signal recognition particle-docking protein FtsY	1.40	4.3E-18	127	532	66	287
Cthe_0770	ffh	signal recognition particle protein	1.17	3.8E-04	173	633	61	231
Cthe_1385	SecA	Protein translocase subunit secA	1.22	4.1E-16	167	615	29	110
Cthe_0903	SecF	protein-export membrane protein SecF	2.24	1.0E-25	119	907	59	463
Cthe_0904	SecD	protein-export membrane protein SecD	2.79	3.1E-63	347	3859	124	1411
Cthe_2718	SecE	preprotein translocase, SecE subunit	2.58	1.1E-33	33	314	64	635
Cthe_0144	SecG	preprotein translocase, SecG subunit	1.35	3.3E-09	254	1043	478	2023
Cthe_2923	SecY	preprotein translocase, SecY subunit	2.28	1.8E-44	557	4307	206	1646

			log2	Signif.	Raw gene reads		FPKM	
**Locus tag**	**Syn.**	**Product (GenBank genome CP000568.1)**	**(fold.diff.)**	**p**	**PL**	**SS**	**PL**	**SS**
Cthe_0957	YajC	preprotein translocase, YajC subunit	2.49	1.7E-54	107	954	169	1568
Cthe_2367	YidC	membrane protein insertase, YidC/Oxa1 family	1.46	1.3E-22	228	999	124	559
**Signal peptidases**								
Cthe_0350		signal peptidase I	1.76	6.3E-22	84	453	68	381
Cthe_0764		signal peptidase I	0.18	4.2E-01	128	232	92	171
Cthe_0909		Lipoprotein signal peptidase	1.80	5.4E-15	24	132	25	142
Cthe_1330		signal peptidase I	2.00	5.2E-27	124	795	112	740
Cthe_1421		signal peptide peptidase SppA, 36K type	2.04	9.1E-26	62	412	30	206
Cthe_2079		signal peptidase I	0.21	2.8E-01	337	619	282	535
**Section 12.**								
**Cell division proteins (membrane associated)**								
**ABC-2-type cell division proteins (FtsX,E and Y)**								
Cthe_1858	FtsY	Peptidase M23	1.67	4.8E-20	182	911	76	396
Cthe_1859	FtsX	protein of unknown function DUF214	1.66	3.2E-18	111	560	60	310
Cthe_1860	FtsE	cell division ATP-binding protein FtsE	2.27	2.2E-25	40	304	26	210
Cthe_0442	FtsQ	Polypeptide-transport-associated domain protein FtsQ-type	1.23	7.2E-13	125	464	68	264
Cthe_0444	FtsA	cell division protein FtsA	0.84	4.3E-08	173	490	66	194
Cthe_0445	FtsZ	cell division protein FtsZ	0.76	6.2E-05	326	871	136	377
Cthe_0975	SpoVE	cell division protein FtsW	-0.97	2.0E-04	145	115	59	49
Cthe_0980	FtsL	cell division protein FtsL	-2.23	3.1E-67	1267	427	1229	428
Cthe_0638	SpoIIIE	cell divisionFtsK/SpoIIIE	0.63	1.3E-06	330	808	33	85
Cthe_1095	SpoIIIE	cell divisionFtsK/SpoIIIE	-3.09	1.2E-27	2532	451	491	92
Cthe_1968	SpoIIIE	cell divisionFtsK/SpoIIIE	0.08	7.6E-01	245	410	36	63
Cthe_3039	SpoIIIE	cell divisionFtsK/SpoIIIE	0.81	5.4E-06	128	357	24	70
**Section 13.**								
**Polyamines (spermidine and putrescine biosynthesis)**								
Cthe_0715		S-adenosylmethionine decarboxylase proenzyme	1.01	2.2E-02	84	278	107	362
Cthe_0694		Spermidine synthase	0.12	5.7E-01	662	1147	380	679
Cthe_1918		Orn/Lys/Arg decarboxylase major region	0.10	5.2E-01	459	782	149	262
Cthe_2108		Orn/Lys/Arg decarboxylase major region	0.98	2.4E-06	46	143	15	48
Cthe_0695		agmatinase	-0.35	3.3E-02	1109	1365	604	771
**Section 14.**								
**Cell wall components**								
**Fatty acid biosynthesis (initiation and elongation)**								
Cthe_0130	FabH	3-Oxoacyl-[acyl-carrier-protein (ACP)] synthase III domain-containing protein	0.04	8.8E-01	313	508	143	241
Cthe_0132	FabH	3-Oxoacyl-[acyl-carrier-protein (ACP)] synthase III domain-containing protein	1.12	5.1E-06	55	190	25	90
Cthe_0135		Beta-ketoacyl synthase	1.39	3.2E-18	533	2202	31	131
Cthe_0936	FabH	3-oxoacyl-[acyl-carrier-protein] synthase 3	1.17	8.3E-07	240	859	114	422
Cthe_0935	FabD	malonyl CoA-acyl carrier protein transacylase	1.53	3.7E-12	178	820	90	430
Cthe_0932	FabF	3-oxoacyl-[acyl-carrier-protein] synthase 2	2.15	1.1E-64	415	2922	159	1158
Cthe_0934	FabG	3-oxoacyl-(acyl-carrier-protein) reductase	1.03	5.7E-08	281	917	180	603
Cthe_2625	FabZ	(3R)-hydroxymyristoyl-[acyl-carrier-protein] dehydratase	2.01	7.9E-08	13	81	13	90
Cthe_3169	FabI	short-chain dehydrogenase/reductase SDR	2.65	4.6E-58	73	737	46	476
**Glycerophospholipids metabolism (includes phosphatidylglycerol and CDP-diacylglycerol biosynthesis)**								
Cthe_1022		NAD-dependent glycerol-3-phosphate dehydrogenase domain protein	1.21	2.9E-11	99	364	46	175
Cthe_1023		Glycerol-3-phosphate acyltransferase	1.21	1.8E-08	40	147	29	109
Cthe_0937		Phosphate acyltransferase	0.71	1.1E-04	407	1058	189	509
Cthe_0713		1-acyl-sn-glycerol-3-phosphate acyltransferase	0.96	7.8E-07	139	428	107	343
Cthe_1000		phosphatidate cytidylyltransferase	1.85	1.9E-12	30	175	17	102
Cthe_0941		CDP-diacylglycerol/glycerol-3-phosphate 3-phosphatidyltransferase	0.26	2.1E-01	80	150	61	119
Cthe_1259		CDP-alcohol phosphatidyltransferase	1.00	5.3E-11	151	477	132	431
Cthe_1396		phospholipase D/Transphosphatidylase	1.22	3.0E-05	18	65	5	21
Cthe_0030		phosphatidate cytidylyltransferase	-0.31	2.2E-01	45	58	30	40
**Flippase (aid the movement of phospholipids between the two membrane leaflets, i.e., transverse diffusion)**								
Cthe_1051		integral membrane protein MviN	1.75	4.9E-15	30	162	9	50

Locus tag	Syn.	Product (GenBank genome CP000568.1)	log2 (fold.diff.)	Signif. p	Raw gene reads PL	SS	FPKM PL	SS
Cthe_2636		integral membrane protein MviN	-1.75	1.3E-02	6	3	2	1
**Peptidoglycan synthesis**								
Cthe_0441		EPSP synthase (3-phosphoshikimate 1-carboxyvinyltransferase)	0.62	8.7E-04	72	175	40	99
Cthe_0973		EPSP synthase (3-phosphoshikimate 1-carboxyvinyltransferase)	-0.28	7.9E-01	2	3	3	3
Cthe_2328		UDP-N-acetylglucosamine 1-carboxyvinyltransferase	1.49	7.0E-20	109	488	41	190
Cthe_2615		UDP-N-acetylglucosamine 1-carboxyvinyltransferase	-0.45	1.6E-02	337	388	126	150
Cthe_0112	murB	UDP-N-acetylenolpyruvoylglucosamine reductase	0.92	2.1E-06	81	244	42	130
Cthe_2626	murC	UDP-N-acetylmuramate--L-alanine ligase	0.45	3.5E-02	130	283	44	99
Cthe_1041	murD	UDP-N-acetylmuramoylalanine--D-glutamate ligase	0.44	3.3E-03	361	773	122	271
Cthe_0979		penicillin-binding protein transpeptidase	-1.42	1.7E-16	1456	853	316	192
Cthe_0978		UDP-N-acetylmuramyl-tripeptide synthetase	-1.31	1.2E-11	662	415	213	139
Cthe_0977		UDP-N-acetylmuramoylalanyl-D-glutamyl-2,6-diaminopimelate/D-alanyl-D-alanyl ligase	-1.89	4.8E-20	595	249	203	89
Cthe_0976	mraY	Phospho-N-acetylmuramoyl-pentapeptide-transferase	-0.94	2.5E-10	309	254	147	125
Cthe_0974	murG	UDP-N-acetylglucosamine--N-acetylmuramyl-(pentapeptide) pyrophosphoryl-undecaprenol N-acetylglucosamine transferase	-1.57	3.9E-11	653	343	276	151
Cthe_1011		penicillin-binding protein transpeptidase	-3.52	4.8E-18	40	5	11	2
Cthe_3047		penicillin-binding protein transpeptidase	1.09	4.6E-07	50	166	17	58
Cthe_1760	mrcA/B	penicillin-binding protein, 1A family	1.76	2.6E-37	161	864	31	171
Cthe_0091	mrdA	penicillin-binding protein 2	0.73	8.6E-07	204	533	45	123
Cthe_0272		peptidase S11 D-alanyl-D-alanine carboxypeptidase 1	-1.72	1.7E-07	116	55	48	23
Cthe_0679		peptidase S11 D-alanyl-D-alanine carboxypeptidase 1	-1.20	9.6E-04	314	218	124	87
Cthe_1899		peptidase M15B and M15C DD-carboxypeptidase VanY/endolysin	2.15	1.3E-23	71	500	38	281
Cthe_3179		peptidase S11 D-alanyl-D-alanine carboxypeptidase 1	-3.16	1.1E-17	95	16	36	6
Cthe_1231		peptidase S11 D-alanyl-D-alanine carboxypeptidase 1	2.42	4.7E-13	9	76	3	29
Cthe_1171		peptidase S11 D-alanyl-D-alanine carboxypeptidase 1	1.01	2.2E-06	145	471	55	185
Cthe_1938		D-alanine/D-alanine ligase	-0.53	3.2E-03	303	329	126	142
Cthe_2305		Undecaprenyl-diphosphatase	1.28	1.5E-08	38	149	22	89
**S-layer proteins**								
Cthe_1368		S-layer domain-containing protein	0.41	4.5E-02	3711	7860	827	1802
Cthe_2506		S-layer domain-containing protein	-4.64	6.5E-58	5973	361	931	59
Cthe_3122		S-layer domain-containing protein	1.48	3.1E-11	246	1104	56	259
Cthe_2611		S-layer domain-containing protein	-5.11	1.2E-93	855	38	82	4
Cthe_2877		S-layer domain-containing protein	-0.75	3.9E-08	496	470	134	131
Cthe_1931		S-layer domain-containing protein	0.90	1.5E-10	228	672	69	210
Cthe_2384		S-layer domain-containing protein	0.66	1.2E-03	147	370	123	319
Cthe_2613		S-layer domain-containing protein	-1.07	8.0E-08	110	83	60	47
Cthe_1932		S-layer domain-containing protein	-0.64	2.3E-02	40	40	6	7
Cthe_2155		S-layer domain-containing protein	-0.77	3.1E-03	37	35	9	9
Cthe_2782		S-layer domain-containing protein	-2.21	4.2E-07	21	7	13	5
Cthe_1505		S-layer domain-containing protein	-0.15	7.9E-01	8	11	6	8
Cthe_2454		S-layer domain-containing protein	-1.72	3.1E-03	9	4	1	1

Section 15.

Pyrimidine synthesis via L-glutamine and L-aspartate

Purinesynthesis via L-glycine, 5-phosphoribosyl pyrophosphate (PRPP) and L-aspartate

Cthe_0053		ribonucleoside-diphosphate reductase, adenosylcobalamin-dependent	1.28	8.1E-14	508	1971	101	404
Cthe_0375		GMP synthase [glutamine-hydrolyzing]	2.64	1.3E-83	371	3697	115	1178
Cthe_0554		phosphoribosylformylglycinamidine synthase	1.78	3.0E-14	255	1403	32	181
Cthe_0681		IMP dehydrogenase/GMP reductase	1.93	3.6E-27	516	3162	164	1037
Cthe_0712		Cytidylate kinase	0.96	1.3E-05	94	294	66	210
Cthe_0716		nucleoside diphosphate kinase	0.17	6.4E-01	34	62	40	74
Cthe_0741		adenylosuccinate lyase	0.58	3.9E-02	297	710	99	243
Cthe_0947		dihydroorotate dehydrogenase family protein	2.00	5.2E-11	16	105	8	56
Cthe_0949		carbamoyl-phosphate synthase, large subunit	1.98	2.7E-16	277	1722	40	263
Cthe_0950		carbamoyl-phosphate synthase, small subunit	2.40	9.0E-20	66	552	29	253
Cthe_0951		Orotidine 5'-phosphate decarboxylase	2.30	9.5E-16	60	473	30	246
Cthe_0952		dihydroorotase, multifunctional complex type	2.57	1.1E-22	87	823	32	316
Cthe_0953		Aspartate carbamoyltransferase	3.29	2.9E-31	33	513	16	269
Cthe_1004		uridylate kinase	1.52	1.6E-15	141	647	95	449
Cthe_1227		thymidylate synthase	1.23	1.2E-09	59	221	34	130

Locus tag	Syn.	Product (GenBank genome CP000568.1)	log2 (fold.diff.)	Signif. p	Raw gene reads PL	Raw gene reads SS	FPKM PL	FPKM SS
Cthe_1245		Phosphoribosylamine--glycine ligase	1.65	6.3E-18	223	1108	84	431
Cthe_1246		phosphoribosylaminoimidazolecarboxamide formyltransferase/IMP cyclohydrolase	2.03	2.3E-25	163	1057	50	335
Cthe_1247		phosphoribosylglycinamide formyltransferase	1.77	1.1E-21	43	234	32	182
Cthe_1248		phosphoribosylformylglycinamidine cyclo-ligase	1.71	6.2E-23	144	746	67	358
Cthe_1249		amidophosphoribosyltransferase	2.27	2.6E-33	97	739	31	246
Cthe_1250		phosphoribosylaminoimidazole carboxylase, catalytic subunit	2.50	3.9E-24	36	325	33	305
Cthe_1315		guanylate kinase	1.16	3.2E-15	163	579	126	464
Cthe_1867		carbamoyl-phosphate synthase, small subunit	3.45	5.1E-44	112	1972	50	906
Cthe_1868		carbamoyl-phosphate synthase, large subunit	3.43	1.5E-42	258	4477	38	683
Cthe_1923		CTP synthase	1.00	4.5E-05	446	1419	130	427
Cthe_2085		deoxyuridine 5'-triphosphate nucleotidohydrolase Dut	-0.46	1.3E-02	109	125	100	118
Cthe_2107		thymidylate kinase	1.80	1.7E-18	51	281	35	202
Cthe_2885		Phosphoribosylaminoimidazole-succinocarboxamide synthase	1.58	9.1E-24	113	535	60	296
Cthe_2924		adenylate kinase	2.36	3.9E-52	522	4235	379	3184
Cthe_3093		Adenylosuccinate synthetase	0.92	5.6E-10	629	1897	234	729

Section 16.

Folates biosynthesis and salvage

Tetrahydrofolate synthesis via GTP

Locus tag	Syn.	Product (GenBank genome CP000568.1)	log2 (fold.diff.)	Signif. p	Raw gene reads PL	Raw gene reads SS	FPKM PL	FPKM SS
Cthe_2203		GTP cyclohydrolase 1	1.34	2.0E-13	89	362	75	314
Cthe_2582		dihydroneopterin aldolase	1.47	2.1E-08	26	116	34	154
Cthe_2583		2-amino-4-hydroxy-6-hydroxymethyldihydropteridine pyrophosphokinase	1.11	1.4E-05	30	103	27	97
Cthe_2581		dihydropteroate synthase	1.44	1.8E-20	92	395	37	164
Cthe_1226		dihydrofolate reductase region	0.99	6.5E-08	87	276	82	268

Tetrahydrofolate transformations

Locus tag	Syn.	Product (GenBank genome CP000568.1)	log2 (fold.diff.)	Signif. p	Raw gene reads PL	Raw gene reads SS	FPKM PL	FPKM SS
Cthe_1058	glyA	glycine hydroxymethyltransferase	2.77	1.1E-68	384	4145	146	1642
Cthe_0099		5,10-methylenetetrahydrofolate reductase	2.80	2.9E-28	20	224	11	126
Cthe_1247		phosphoribosylglycinamide formyltransferase	1.77	1.1E-21	43	234	32	182
Cthe_2399		Formate--tetrahydrofolate ligase	0.95	1.5E-10	295	912	84	267
Cthe_0645		homocysteine S-methyltransferase	-0.62	5.0E-03	760	782	149	159
Cthe_1093		Tetrahydrofolate dehydrogenase/cyclohydrolase, NAD(P)-binding domain	-1.70	2.5E-12	1481	718	816	409
Cthe_1295		5-formyltetrahydrofolate cyclo-ligase	-1.51	3.6E-18	281	155	231	132

Section 17.

Homologous recombination (RecFOR and partial RecBCD pathways; with RuvABC)

Locus tag	Syn.	Product (GenBank genome CP000568.1)	log2 (fold.diff.)	Signif. p	Raw gene reads PL	Raw gene reads SS	FPKM PL	FPKM SS
Cthe_0208	RecJ	single-stranded-DNA-specific exonuclease RecJ	-0.75	8.9E-05	154	144	32	31
Cthe_1346	RecJ	single-stranded-DNA-specific exonuclease RecJ	1.40	9.5E-16	132	551	25	108
Cthe_1350	SSB	single-strand binding protein	-0.49	5.2E-03	183	205	214	249
Cthe_2186	SSB	single-strand binding protein	2.61	4.4E-48	311	3052	355	3589
Cthe_2249	RecD	helicase, RecD/TraA family	0.14	4.7E-01	113	196	24	43
Cthe_3190	RecD	helicase, RecD/TraA family	1.02	1.7E-14	199	637	44	147
Cthe_2374	RecF	DNA replication and repair protein recF	1.25	4.5E-12	80	302	34	133
Cthe_1050	RecA	Protein recA	-0.22	2.2E-01	609	822	274	383
Cthe_1066	RecO	DNA repair protein recO	-0.36	8.2E-02	90	111	56	72
Cthe_2142	RecR	Recombination protein recR	1.79	2.1E-26	165	914	131	747
Cthe_0181	RuvA	Holliday junction ATP-dependent DNA helicase ruvA	1.59	1.1E-09	27	129	21	103
Cthe_0182	RuvB	Holliday junction ATP-dependent DNA helicase ruvB	1.38	3.4E-15	61	254	29	126
Cthe_0180	RuvC	Crossover junction endodeoxyribonuclease ruvC	2.26	1.4E-19	18	136	17	136
Cthe_1278	RecG	ATP-dependent DNA helicase RecG	0.31	6.4E-02	120	237	27	56

DNA polymerases

Locus tag	Syn.	Product (GenBank genome CP000568.1)	log2 (fold.diff.)	Signif. p	Raw gene reads PL	Raw gene reads SS	FPKM PL	FPKM SS
Cthe_0566	PriA	primosomal protein N'	-0.92	2.8E-12	821	685	159	137
Cthe_0886	DpoI	DNA polymerase I	1.30	1.8E-20	166	647	29	118
Cthe_1650	DpoI	DNA-directed DNA polymerase	-2.51	1.2E-02	4	1	1	0
Cthe_0996	polC	DNA polymerase III, alpha subunit	0.67	1.1E-04	294	739	32	84
Cthe_1040	DpoIII	DNA polymerase III, delta subunit	0.74	7.7E-05	64	172	30	82
Cthe_1264	dnaE	DNA polymerase III, alpha subunit	-2.29	1.1E-39	2100	670	281	93
Cthe_2105	DpoIII	AAA ATPase	-2.37	4.3E-52	1257	378	600	188
Cthe_2144	DpoIII	DNA polymerase III, subunits gamma and tau	1.24	1.7E-09	130	485	37	144
Cthe_2373	DpoIII	S4 domain protein YaaA	1.67	8.0E-08	15	76	34	178

Section 18.

Locus tag	Syn.	Product (GenBank genome CP000568.1)	log2 (fold.diff.)	Signif. p	Raw gene reads PL	Raw gene reads SS	FPKM PL	FPKM SS
**Chemotaxis**								
Cthe_0288		MCP methyltransferase CheR-type	2.34	2.7E-21	30	242	13	106
Cthe_0717		MCP methyltransferase CheR-type	-0.61	1.2E-01	96	102	59	64
Cthe_0808		MCP methyltransferase CheR-type	-2.15	2.3E-06	21	7	12	4
Cthe_2282		MCP methyltransferase CheR-type	-1.93	2.1E-03	9	4	5	2
Cthe_2820		MCP methyltransferase CheR-type	-0.64	1.9E-01	11	11	6	6
Cthe_0039		chemotaxis sensory transducer	-1.55	8.7E-05	24	13	6	3
Cthe_0298		chemotaxis sensory transducer	-0.51	9.2E-02	37	41	10	11
Cthe_2663		chemotaxis sensory transducer	-2.01	1.8E-19	410	159	155	62
Cthe_2819		chemotaxis sensory transducer	-0.55	2.8E-03	386	412	41	46
Cthe_3156		chemotaxis sensory transducer	0.70	8.0E-04	244	625	92	245
Cthe_0401		histidine kinase HAMP region domain protein	-3.91	5.9E-83	4198	429	874	93
Cthe_0489		Chemotaxis response regulator protein-glutamate methylesterase	-1.59	3.5E-18	263	137	117	63
Cthe_0807		Chemotaxis response regulator protein-glutamate methylesterase	-1.81	2.8E-05	28	12	12	6
Cthe_2281		Chemotaxis response regulator protein-glutamate methylesterase	-0.69	9.9E-02	16	15	7	7
Cthe_2821		Chemotaxis response regulator protein-glutamate methylesterase	-0.33	3.4E-01	26	33	11	15
Cthe_0493		Chemoreceptor glutamine deamidase cheD	-1.80	6.5E-17	137	62	133	62
Cthe_0490		CheW domain protein	-1.49	6.1E-14	677	374	154	89
Cthe_0810		ATP-binding region ATPase domain protein	-1.90	2.8E-08	59	25	13	6
Cthe_2284		ATP-binding region ATPase domain protein	-1.02	5.6E-04	45	35	10	8
Cthe_2818		CheW domain protein	-0.06	8.1E-01	140	212	37	57
Cthe_0491		CheW domain protein	-1.51	2.7E-11	145	79	147	84
Cthe_0809		CheW domain protein	-2.42	2.9E-05	17	5	17	5
Cthe_0878		CheW domain protein	-0.38	7.3E-02	557	672	453	571
Cthe_2285		CheW domain protein	0.33	6.5E-01	4	7	4	8
Cthe_2817		CheW domain protein	0.17	6.9E-01	17	31	19	35
Cthe_3029		CheW domain protein	-3.28	1.5E-05	10	1	9	1
Cthe_0479		response regulator receiver	-1.95	2.0E-26	326	133	427	180
Cthe_0805		response regulator receiver	-2.93	2.6E-05	15	3	17	3
Cthe_0492		CheC domain protein	-1.23	1.2E-06	71	47	54	38
Cthe_0126		CheC domain protein	-3.37	9.5E-07	12	2	12	2
Cthe_2308		CheC domain protein	-3.11	9.7E-07	12	2	12	2

Section 19.

Flagellar assembly and swimming motility

Locus tag	Syn.	Product (GenBank genome CP000568.1)	log2 (fold.diff.)	Signif. p	Raw gene reads PL	Raw gene reads SS	FPKM PL	FPKM SS
Cthe_2425	MotA	MotA/TolQ/ExbB proton channel	0.35	6.1E-01	5	9	2	5
Cthe_2426	MotB	OmpA/MotB domain protein	0.14	7.7E-01	10	18	6	11
Cthe_0462	FlgB	flagellar basal-body rod protein FlgB	-0.05	8.3E-01	145	224	170	270
Cthe_0463	FlgC	flagellar basal-body rod protein FlgC	-0.50	9.4E-03	143	160	153	177
Cthe_0464	FliE	flagellar hook-basal body complex subunit FliE	-0.60	2.3E-02	46	47	68	74
Cthe_0465	FliF	flagellar M-ring protein FliF	-0.91	3.4E-10	258	217	79	68
Cthe_0466	FliG	flagellar motor switch protein FliG	-0.75	4.0E-04	161	151	75	73
Cthe_0467	FliH	Flagellar assembly protein FliH/Type III secretion system HrpE	-1.34	3.7E-08	133	83	80	51
Cthe_0468	FliI	flagellar protein export ATPase FliI	-1.62	1.7E-04	75	37	27	14
Cthe_0469	FliJ	flagellar export protein FliJ	-2.66	2.6E-14	55	13	56	14
Cthe_0470	FlbB	hypothetical protein	-1.37	3.7E-05	30	18	17	10
Cthe_0471	FliK	flagellar hook-length control protein	-1.17	1.3E-11	185	129	55	40
Cthe_0472	FlgD	flagellar hook capping protein	-1.30	1.0E-05	82	52	40	26
Cthe_0473		flagellar operon protein	-1.21	8.7E-04	23	16	28	20
Cthe_0474	FlgE	fagellar hook-basal body protein	-2.40	4.7E-22	406	118	145	44
Cthe_0475		flagellar FlbD family protein	-0.96	1.1E-04	54	44	88	74
Cthe_0476	FliL	flagellar basal body-associated protein FliL	-1.70	2.3E-11	338	162	319	159
Cthe_0477	FliM	flagellar motor switch protein FliM	-1.85	1.1E-17	1130	488	544	244
Cthe_0478	FliN	flagellar motor switch protein FliN	-1.61	9.3E-13	968	495	377	199
Cthe_0480	FliO	hypothetical protein	-1.40	3.3E-17	369	220	352	218
Cthe_0481	FliP	flagellar biosynthetic protein FliP	-1.50	4.8E-16	183	102	111	64
Cthe_0482	FliQ	flagellar biosynthetic protein FliQ	-1.26	3.1E-12	123	81	217	147
Cthe_0483	FliR	flagellar biosynthetic protein FliR	-1.37	6.9E-20	217	133	131	83
Cthe_0484	FlhB	flagellar biosynthetic protein FlhB	-1.70	1.9E-24	694	333	278	139
Cthe_0485	FlhA	flagellar biosynthesis protein FlhA	-1.42	5.3E-19	591	347	138	84
Cthe_0486	FlhF	flagellar biosynthetic protein FlhF	-1.43	1.9E-18	450	260	175	106
Cthe_0487	FlhG	Cobyrinic acid ac-diamide synthase	-1.63	8.3E-16	213	108	111	59
Cthe_2216	FlgN	FlgN family protein	-4.12	1.4E-115	498	45	483	45

Locus tag	Syn.	Product (GenBank genome CP000568.1)	log2 (fold.diff.)	Signif. p	Raw gene reads PL	Raw gene reads SS	FPKM PL	FPKM SS
Cthe_2217	FliS	flagellar protein FliS	-4.32	2.0E-57	172	13	201	16
Cthe_2218	FliD	flagellar hook-associated 2 domain-containing protein	-4.12	3.3E-36	1399	119	264	24
Cthe_2219	FlaG	flagellar protein FlaG protein	-1.02	3.3E-02	12	9	14	11
Cthe_2235		protein of unknown function DUF115	-2.09	1.8E-04	12	4	3	1
Cthe_2236	FliC	flagellin domain protein	-2.37	1.4E-05	15	4	9	3
Cthe_2237	FliC	flagellin domain protein	-1.55	3.9E-03	11	6	6	3
Cthe_2240	fliW	Flagellar assembly factor fliW	-2.64	1.2E-34	206	52	204	53
Cthe_2242	FlgL	flagellar hook-associated protein 3	-0.06	8.8E-01	18	28	10	15
Cthe_2243	FlgK	flagellar hook-associated protein FlgK	-0.24	4.3E-01	34	45	10	14
Cthe_2244	FlgK	flagellar hook-associated protein FlgK	-0.91	5.1E-04	45	38	14	13
Cthe_2245	FlgN	FlgN family protein	-1.25	2.0E-03	18	12	17	12
Cthe_2246	FlgM	flagellar biosynthesis anti-sigma factor protein FlgM	-1.43	2.2E-10	132	76	213	128
Cthe_2620	FlgG	flagellar hook-basal body protein	1.90	1.2E-02	2	10	1	6
Cthe_2621	FlgG	flagellar hook-basal body protein	1.57	6.9E-03	3	16	2	10
Cthe_2622	FlgJ	Flagellar protein FlgJ	1.00	1.0E-05	147	472	200	659
**Pili assembly and twiching motility**								
Cthe_0108	PilZ	type IV pilus assembly PilZ	-1.16	4.8E-10	320	225	237	173
Cthe_0184	PilZ	type IV pilus assembly protein PilM	-1.77	2.6E-16	5089	2345	2177	1042
Cthe_0488		type IV pilus assembly PilZ	-1.59	1.6E-17	129	68	91	49
Cthe_0656		type IV pilus assembly protein PilM	-0.12	3.6E-01	1249	1822	535	807
Cthe_0697		type IV pilus assembly PilZ	-2.25	1.5E-14	170	56	109	37
Cthe_0733		type IV pilus assembly PilZ	-3.88	9.9E-31	1379	142	995	107
Cthe_0868		type IV pilus assembly PilZ	0.74	1.1E-05	170	454	114	313
Cthe_0888		type IV pilus assembly PilZ	-2.30	6.4E-72	1137	365	767	255
Cthe_1065		type IV pilus assembly PilZ	-1.08	3.1E-06	992	733	712	547
Cthe_1173		type IV pilus assembly PilZ	-2.10	7.9E-40	637	234	467	177
Cthe_1865		type IV pilus assembly PilZ	1.95	4.5E-29	468	2898	335	2139
Cthe_2684		type IV pilus assembly PilZ	-3.26	4.5E-34	1667	266	1527	254
Cthe_2686		type IV pilus assembly protein PilM	-0.16	2.9E-01	1556	2199	666	975
Cthe_2893		type IV pilus assembly PilZ	0.44	5.8E-02	164	346	73	161
Cthe_0257	PilT	twitching motility protein	-1.47	4.9E-03	13	7	6	3
Cthe_1106	PilT	twitching motility protein	0.45	4.5E-03	1028	2221	463	1034

Section 20.

Cellulosome and free hydrolytic enzymes

Cellulosomal cellulase enzymes

Cthe_2089	CelS	Glycoside hydrolase family 48	-0.77	1.8E-04	50123	46328	10739	10231
Cthe_0821	CtMan5/	Glycoside hydrolase family 5 / Coagulation factor 41767 type domain protein / Dockerin type 1	-0.53	1.4E-02	26053	28236	7388	8299
Cthe_0412	CelK	Glycoside hydrolase family 9	-0.58	1.8E-02	24367	25899	4338	4730
Cthe_0413	CbhA	Glycoside hydrolase family 9 / Cellulose 1,4-beta-cellobiosidase	-1.80	6.5E-20	15963	7210	2070	965
Cthe_0269	CelA	Glycoside hydrolase family 8 / Dockerin type 1	0.09	6.6E-01	4666	7870	1549	2693
Cthe_0624	CelJ	Glycoside hydrolase family 9 / Xyloglucan-specific exo-beta-1,4-glucanase / non-processive endocellulase	1.28	5.0E-22	2790	10773	276	1099
Cthe_1838	XynC	Glycoside hydrolase family 10 / Endo-1,4-beta-xylanase	0.85	3.7E-11	2335	6702	597	1770
Cthe_1398	Xgh74A	Dockerin type 1	-1.53	2.2E-16	4194	2287	788	444
Cthe_0543	CelF	Glycoside hydrolase family 9	-0.47	1.3E-02	3237	3712	696	822
Cthe_2872	CelG	Glycoside hydrolase family 5	-0.06	6.5E-01	2477	3778	692	1089
Cthe_0625	CelQ	Glycoside hydrolase family 9	0.53	1.3E-02	1826	4192	408	964
Cthe_0536	CelB	Glycoside hydrolase family 5	0.35	1.9E-03	1840	3725	517	1078
Cthe_0578	CelR	Glycoside hydrolase family 9	0.61	1.0E-03	1458	3549	315	789
Cthe_0433		Glycoside hydrolase family 9	-0.52	4.6E-04	2056	2281	413	472
Cthe_2972	XynA	Glycoside hydrolase family 11 / xylanase/chitin deacetylase	-1.16	7.2E-11	2220	1560	512	374
Cthe_1806		Ig domain protein group 2 domain protein	-0.54	2.1E-02	1751	1872	127	141
Cthe_2812	CelT	Glycoside hydrolase family 9	-0.18	3.9E-01	1209	1699	314	454
Cthe_0745	CelW	Glycoside hydrolase family 9	-0.44	9.5E-03	1140	1323	247	296
Cthe_2760	CelV	Glycoside hydrolase family 9	-0.53	3.2E-05	1048	1152	172	196
Cthe_0190		Proteinase inhibitor I4 serpin	-1.90	3.2E-11	1380	582	367	159
Cthe_2193	CtXyl5A	Carbohydrate binding family 6	-0.71	1.2E-05	941	909	157	156
Cthe_1963	XynZ	Glycoside hydrolase family 10	-1.58	1.3E-36	1083	571	204	111
Cthe_0912	XynY	Glycoside hydrolase family 10 / Beta-1,4-xylanase	-3.46	5.0E-38	1243	173	183	26
Cthe_0246		Carbohydrate binding family 6	-1.93	9.8E-30	1017	417	196	83

Locus tag	Syn.	Product (GenBank genome CP000568.1)	log2 (fold.diff.)	Signif. p	Raw gene reads PL	Raw gene reads SS	FPKM PL	FPKM SS
Cthe_2811	ManA	Glycoside hydrolase family 26 / Dockerin type 1	-1.55	4.6E-12	902	483	242	134
Cthe_0270	ChiA	Glycoside hydrolase family 18	-1.34	2.8E-13	833	519	273	175
Cthe_3132		Dockerin type 1	-1.72	3.1E-27	825	390	315	155
Cthe_0825	CelD	Glycoside hydrolase family 9	-0.93	1.7E-04	670	554	164	140
Cthe_0032	CtManF	Glycoside hydrolase family 26 / Dockerin type 1	-0.95	7.0E-09	658	536	176	149
Cthe_0043	CelN	Glycoside hydrolase family 9	-0.53	2.3E-03	580	637	124	140
Cthe_0015	CtGH43	Alpha-L-arabinofuranosidase B	-3.88	9.5E-58	916	96	204	22
Cthe_0274	CelP	Glycoside hydrolase family 9	-0.59	9.5E-04	581	611	163	177
Cthe_0405	CelL	Glycoside hydrolase family 5	0.04	8.5E-01	474	767	142	239
Cthe_0191		Proteinase inhibitor I4 serpin	-2.34	2.9E-23	710	220	188	60
Cthe_2761	LecA	Glycoside hydrolase family 9	-0.45	1.3E-02	447	511	99	118
Cthe_2147	CelO	Glycoside hydrolase family 5	-1.27	6.1E-18	537	353	129	87
Cthe_0435	Doc435	Dockerin type 1	0.00	9.9E-01	356	563	160	262
Cthe_1472	CelH	Glycoside hydrolase family 5 / Carbohydrate-binding family 11	-1.46	5.1E-18	486	277	85	50
Cthe_0797	CelE	Glycoside hydrolase family 5	-1.84	2.5E-19	473	207	92	42
Cthe_0239		Spore coat protein CotH	-1.06	2.4E-05	377	281	57	44
Cthe_2590	XynD	Glycoside hydrolase, family 10 / Carbohydrate-binding, CenC-like / Dockerin type 1	-1.45	2.0E-14	352	202	86	51
Cthe_0258	Doc258	Dockerin type 1	0.00	9.9E-01	236	374	79	130
Cthe_0661	Ct1, 3Ga	Ricin B lectin	-0.63	2.2E-04	269	274	74	79
Cthe_1271	CtAraf;C	Glycoside hydrolase family 43/ Carbohydrate-binding family 6 / Dockerin type 1	-0.73	5.8E-06	270	258	63	62
Cthe_3141		Carbohydrate binding family 6	-1.88	9.1E-17	324	138	62	27
Cthe_0729		Dockerin type 1	-0.44	9.3E-02	193	227	57	69
Cthe_0640		Dockerin type 1	-0.83	9.1E-05	189	169	51	47
Cthe_0211	LicB	Glycoside hydrolase family 16	-0.54	1.5E-03	167	181	79	88
Cthe_3012	CtXynGI	Carbohydrate binding family 6	-1.38	2.5E-17	171	104	43	27
Cthe_1273		Alpha-L-arabinofuranosidase B	-1.14	1.7E-06	145	102	47	34
Cthe_1400		glycosyl hydrolase 53 domain protein	-0.47	5.3E-03	115	131	44	51
Cthe_1890		Dockerin type 1	-2.62	2.0E-16	153	38	34	9
Cthe_3136	CprA	Peptidase S8 and S53 subtilisin kexin sedolisin	1.35	1.6E-11	37	149	16	65
Cthe_2038		Dockerin type 1	-1.25	1.4E-09	88	58	17	12
Cthe_0798	CelX	Lipolytic protein G-D-S-L family	-0.69	2.8E-03	75	73	22	23
Cthe_0660		Glycoside hydrolase family 81 / Dockerin type 1	-3.08	5.0E-15	95	17	20	4
Cthe_2194		Carbohydrate binding family 6	-3.51	7.3E-36	98	13	31	4
Cthe_2950		Pectate lyase/Amb allergen	-0.42	1.8E-01	44	52	12	15
Cthe_2139		Alpha-L-arabinofuranosidase B	-0.65	9.7E-03	46	46	7	8
Cthe_2179		Pectate lyase/Amb allergen	-0.69	8.8E-02	33	32	6	6
Cthe_0044	CseP	Spore coat protein CotH	-0.52	1.6E-01	27	30	8	9
Cthe_2137		Dockerin type 1 protein / Carbohydrate-binding family 6	-2.47	1.3E-08	36	10	7	2
Cthe_2195		Carbohydrate binding family 6	-3.59	1.0E-14	35	4	6	1
Cthe_2879		Dockerin type 1	-0.25	4.8E-01	20	27	6	9
Cthe_2949		Pectinesterase; Dockerin type 1	-1.85	6.5E-05	20	8	5	2
Cthe_2138		Glycoside hydrolase family 43	-0.31	5.1E-01	10	13	3	4
Cthe_2549		Dockerin type 1	-0.87	7.1E-02	11	9	5	4
Cthe_2271		Dockerin type 1	-2.44	3.6E-04	10	3	8	2
Cthe_2196		Glycoside hydrolase family 43 / Dockerin type 1	-3.56	2.7E-04	6	1	2	0
**Non-catalytic cellulosomal proteins**								
Cthe_3077	CipA	Primary cellulosome scaffoldin (type I cohesin and X-dockerin dyad)	-0.23	2.6E-01	101205	137084	8660	12093
Cthe_3078	OlpB	Cellulosome anchoring scaffoldin (type II cohesin)	-1.61	6.9E-17	54048	27912	3715	1976
Cthe_3079	Orf2p	Cellulosome anchoring scaffoldin (type II cohesin)	-1.84	1.6E-16	9714	4257	2236	1010
Cthe_3080	OlpA	Anchoring scaffoldin (type I cohesin)	-2.27	2.0E-34	6797	2215	2409	810
Cthe_0736	ScaE	Anchoring cohesin	0.48	1.9E-02	1814	4050	220	506
Cthe_1307	SdbA	Cellulosome anchoring scaffoldin (type II cohesin)	-0.02	9.2E-01	871	1357	218	351
Cthe_0735		anchoring protein cohesin region	1.40	1.9E-04	83	355	49	215
Cthe_0452	OlpC	Anchoring scaffoldin (type I cohesin)	1.66	2.1E-16	44	220	27	139
**Non-cellulosomal, free cellulase enzymes**								
Cthe_2809	LicA	glycoside hydrolase family 16	-2.86	2.3E-76	8579	1841	1024	228
Cthe_0275	Cbp	glycosyltransferase 36	2.28	2.1E-50	648	4981	126	1003
Cthe_1911		Carbohydrate binding family 6	1.83	1.4E-27	673	3808	83	481
Cthe_2989	Cdp	glycosyltransferase 36	2.36	1.6E-93	452	3712	73	615
Cthe_0271		type 3a cellulose-binding domain protein	-0.60	3.2E-04	1147	1209	588	638
Cthe_1256	bglB	glycoside hydrolase family 3 domain protein	-4.12	2.6E-115	1402	125	293	27
Cthe_3163		Carbohydrate binding family 25	1.10	1.4E-09	427	1460	413	1458

Locus tag	Syn.	Product (GenBank genome CP000568.1)	log2 (fold.diff.)	Signif. p	Raw gene reads PL	SS	FPKM PL	SS
Cthe_0212	bglA	beta-galactosidase	0.91	2.2E-08	462	1375	155	476
Cthe_2119		glycoside hydrolase family 10	-1.34	4.1E-12	672	416	140	89
Cthe_1777		amidohydrolase	1.57	1.0E-21	189	890	74	363
Cthe_1787		glycoside hydrolase 15-related	-0.70	1.5E-03	459	443	113	113
Cthe_2807	CelC	glycoside hydrolase family 5	-2.78	3.7E-35	643	145	294	69
Cthe_2191		1,4-alpha-glucan-branching enzyme	1.04	8.1E-09	169	553	36	123
Cthe_0040	CelI	glycoside hydrolase family 9	1.17	5.3E-16	158	560	28	103
Cthe_0795		alpha amylase catalytic region	-1.97	4.3E-31	371	150	102	43
Cthe_2895		glycoside hydrolase family 18	1.04	1.2E-08	122	398	33	111
Cthe_0071	CelY	glycoside hydrolase family 48	0.37	7.4E-02	147	302	25	53
Cthe_1471		glycoside hydrolase family 5	-2.54	1.1E-47	272	73	76	21
Cthe_3063		Acetyl xylan esterase	1.16	1.1E-09	48	169	23	86
Cthe_0884		Lytic transglycosylase catalytic	0.19	5.0E-01	47	85	40	74
Cthe_2548	CtAraf51	alpha-L-arabinofuranosidase domain protein	0.36	1.2E-01	40	82	13	27
Cthe_0322		glycoside hydrolase family 3 domain protein	0.37	1.2E-01	35	71	12	26
Cthe_1800		Peptidoglycan-binding lysin domain	-1.02	1.3E-02	50	39	16	13
Cthe_2190		N-acetylglucosamine-6-phosphate deacetylase	-0.63	2.7E-02	32	33	13	13
Cthe_1428		glycoside hydrolase family 1	-1.15	6.2E-03	18	13	6	5
Cthe_1613		glycosyl hydrolase-like protein protein	-2.45	6.1E-06	13	4	3	1
Cthe_2744		Lytic transglycosylase catalytic	0.65	2.2E-01	5	13	4	9
**Section 21.**								
**Redox homeostatis and stress response**								
**Methionine repair**								
Cthe_2990	msrA	Peptide methionine sulfoxide reductase msrA	-3.93	2.2E-31	223	22	178	18
**Antioxidants (di-sulfur bridge)**								
Cthe_0360		thioredoxin	-5.20	7.4E-59	36792	1476	53059	2230
Cthe_0235		glutaredoxin-like protein, YruB-family	-4.32	1.3E-53	4516	341	9346	737
Cthe_1965		peroxiredoxin	-4.96	2.1E-62	57232	2743	48277	2413
Cthe_1465		alkyl hydroperoxide reductase/ Thiol specific antioxidant/ Mal allergen	-3.93	2.4E-22	1489	141	1566	156
Cthe_1947		alkyl hydroperoxide reductase/ Thiol specific antioxidant/ Mal allergen	-3.82	2.9E-129	2597	287	2063	236
Cthe_1964		alkyl hydroperoxide reductase, F subunit	-5.75	2.0E-144	70270	1998	21790	641
Cthe_0173		FAD-dependent pyridine nucleotide-disulphide oxidoreductase	-1.76	1.6E-03	10	5	4	2
Cthe_0200		FAD-dependent pyridine nucleotide-disulphide oxidoreductase	-3.18	9.4E-16	1027	165	394	67
Cthe_0560		FAD-dependent pyridine nucleotide-disulphide oxidoreductase	-4.95	2.4E-33	19384	886	5183	249
Cthe_1164		FAD-dependent pyridine nucleotide-disulphide oxidoreductase	1.34	4.0E-19	123	492	37	152
Cthe_1945		FAD-dependent pyridine nucleotide-disulphide oxidoreductase	-2.35	7.6E-82	1492	462	790	253
Cthe_1946		FAD-dependent pyridine nucleotide-disulphide oxidoreductase	-2.65	1.3E-110	3435	862	1266	329
Cthe_3004		FAD-dependent pyridine nucleotide-disulphide oxidoreductase	1.45	6.0E-13	195	840	62	278
**Antioxidants (iron-sulfur cluster)**								
Cthe_0063		Rubredoxin-type Fe(Cys)4 protein	-5.06	1.4E-72	19746	883	27880	1303
Cthe_2164		Rubredoxin-type Fe(Cys)4 protein	-2.67	1.5E-25	1563	375	4655	1166
**Other putative antioxidants**								
Cthe_1948		cytochrome c biogenesis protein transmembrane region	-3.23	3.7E-23	1741	275	1233	205
Cthe_1509		protein of unknown function DUF438	-6.13	5.9E-70	14333	293	5484	118
**Copper sequestration and transport**								
Cthe_0738		copper ion binding protein	-4.24	1.9E-48	3157	249	7057	584
Cthe_1848		copper-translocating P-type ATPase	-5.70	1.7E-84	8051	229	1703	51
**Section 22.**								
**Riboflavin, FMN and FAD synthesis**								
Cthe_0104		riboflavin biosynthesis protein RibD	-3.50	1.6E-37	4871	654	2100	294
Cthe_0105		riboflavin synthase, alpha subunit	-3.93	3.0E-66	942	95	678	71

Locus tag	Syn.	Product (GenBank genome CP000568.1)	log2 (fold.diff.)	Signif. p	Raw gene reads PL	Raw gene reads SS	FPKM PL	FPKM SS
Cthe_0106		GTP cyclohydrolase-2	-3.27	9.4E-53	3148	507	1198	200
Cthe_0107		6,7-dimethyl-8-ribityllumazine synthase	-2.69	1.8E-31	545	132	550	138
Cthe_0987		riboflavin biosynthesis protein RibF	0.06	7.8E-01	310	513	156	267
**Section 23.**								
**Fe-S cluster repair (Nif)**								
Cthe_0720	NifS	Cysteine desulfurase	-4.62	2.1E-65	40392	2463	16112	1030
Cthe_0721	NifU	FeS cluster assembly scaffold protein NifU	-4.99	4.7E-194	4560	226	4803	246
**tRNA-thiolation**								
Cthe_0722	Mnma	tRNA-specific 2-thiouridylase mnmA	-4.08	6.3E-113	5476	507	2397	229
**Section 24.**								
**Assimilatory sulfate reduction (sulfate to sulfite)**								
Cthe_2535		adenylylsulfate reductase, thioredoxin dependent	-2.44	1.7E-03	6	2	4	1
Cthe_2536		phosphoadenosine phosphosulfate reductase	-2.10	1.8E-08	36	13	19	7
Cthe_2537		sulfate adenylyltransferase, large subunit	-1.70	9.2E-05	23	11	6	3
**Siroheme synthesis (siroheme converts sulfite to sulfide)**								
Cthe_2525		Glutamyl-tRNA reductase	-2.79	1.7E-07	17	4	8	2
Cthe_2526		precorrin-6X reductase	-3.57	1.2E-07	13	2	15	2
Cthe_2527		Porphobilinogen deaminase	-2.17	1.9E-05	14	5	8	3
Cthe_2528		uroporphyrin-III C-methyltransferase	-2.47	1.4E-10	27	8	9	3
Cthe_2529		delta-aminolevulinic acid dehydratase	-1.60	2.6E-03	15	7	7	4
Cthe_2530		glutamate-1-semialdehyde-2,1-aminomutase	-2.18	6.3E-05	15	5	5	2
**Section 25.**								
**Indole synthesis (Tryptophan precursor)**								
Cthe_0874		glutamine amidotransferase of anthranilate synthase	-2.49	1.7E-46	1227	342	960	277
Cthe_0875		anthranilate synthase component I	-2.24	1.8E-38	4009	1324	1279	439
Cthe_0873		Anthranilate phosphoribosyltransferase	-2.41	1.9E-38	4006	1167	1827	554
Cthe_0871		N-(5'phosphoribosyl)anthranilate isomerase (PRAI)	-2.92	1.6E-67	3315	680	2380	509
Cthe_0872		Indole-3-glycerol phosphate synthase	-2.48	4.3E-57	2006	559	1212	352
**Section 26.**								
**Exinucleases (Nucleotide exision repair (NER) of damaged DNA)**								
Cthe_0309	UvrB	excinuclease ABC, B subunit	-1.03	7.0E-12	911	703	217	173
Cthe_0311	UvrA	excinuclease ABC, A subunit	-3.77	9.0E-138	5108	581	853	101
Cthe_2737	UvrC	UvrABC system protein C	-5.38	1.6E-52	10618	379	2663	99
Cthe_0206	UvrD	UvrD/REP helicase	-1.26	1.7E-06	242	157	51	34
Cthe_0968	UvrD	UvrD/REP helicase	-0.68	1.4E-01	51	50	11	12
**Section 27.**								
**Proteases (degradation of abnormal proteins)**								
**LON protease**								
Cthe_0082	LonA	ATP-dependent protease La	-4.72	3.2E-90	16310	941	3143	189
Cthe_2742	LonB	ATP-dependent protease LonB	-2.58	5.8E-23	72	19	20	5
**CLP proteases**								
Cthe_1216	ClpS	ATP-dependent Clp protease adapter protein clpS	-0.24	2.6E-01	82	109	130	179
Cthe_1217	ClpA	ATP-dependent Clp protease, ATP-binding subunit clpA	-0.31	3.3E-02	603	769	122	162
Cthe_2740	ClpP	ATP-dependent Clp protease proteolytic subunit	-3.36	1.9E-50	23649	3514	19130	2971
Cthe_2741	ClpX	ATP-dependent Clp protease ATP-binding subunit clpX	-3.04	1.7E-135	15229	2903	5549	1099
Cthe_1789		ATPase AAA-2 domain protein	-3.86	2.4E-181	7336	787	1412	157
Cthe_0312	ClpB	ATPase AAA-2 domain protein	-1.39	2.5E-16	1370	820	268	166
**Metalloprotease (damaged membrane protein disassembly)**								
Cthe_2253	FtsH	ATP-dependent metalloprotease FtsH	-1.82	3.0E-63	4372	1960	1149	533
**Section 28.**								
**Sporulation**								
Cthe_0044		Spore coat protein CotH	-0.52	1.6E-01	27	30	8	9
Cthe_0083		Stage II sporulation protein E	-0.13	6.8E-01	65	95	18	27
Cthe_0116		sporulation transcription regulator whiA	0.54	1.4E-03	126	289	63	150
Cthe_0122		stage V sporulation protein AC	-0.72	4.3E-01	3	3	3	3
Cthe_0123		stage V sporulation protein AD	-1.59	9.2E-03	8	4	4	2
Cthe_0124		stage V sporulation protein AE	-2.98	4.8E-04	6	1	8	1
Cthe_0125		stage V sporulation protein ae	-3.17	9.5E-18	54	9	45	8
Cthe_0187		Lipoprotein LpqB, GerMN domain	-0.42	4.0E-02	315	376	128	157

Locus tag	Syn.	Product (GenBank genome CP000568.1)	log2 (fold.diff.)	Signif. p	Raw gene reads PL	SS	FPKM PL	SS
Cthe_0216		small acid-soluble spore protein alpha/beta type	0.39	4.7E-01	8	17	14	29
Cthe_0239		Spore coat protein CotH	-1.06	2.4E-05	377	281	57	44
Cthe_0280		Coat F domain protein	-0.02	9.8E-01	6	10	9	15
Cthe_0300		Sporulation uncharacterized protein YkwD	-1.79	1.1E-05	24	11	19	9
Cthe_0415		spore coat protein CotJB	-1.56	1.8E-01	2	1	3	1
Cthe_0446		sigma-E processing peptidase SpoIIGA	-1.82	2.1E-06	49	22	26	12
Cthe_0449		sporulation protein, YlmC/YmxH family	-1.43	3.6E-02	9	5	17	10
Cthe_0583		Stage II sporulation protein E	0.91	1.1E-03	42	126	17	52
Cthe_0623		sporulation protein, yteA family	-0.65	1.5E-03	168	168	124	129
Cthe_0641		Coat F domain protein	-2.73	6.5E-05	199	44	398	88
Cthe_0667		GerA spore germination protein	-2.27	9.9E-06	32	10	9	3
Cthe_0668		spore germination protein	-1.09	1.3E-01	5	4	2	2
Cthe_0669		germination protein, Ger(x)C family	-0.91	2.4E-01	6	5	2	2
Cthe_0670		spore germination protein	-2.93	3.3E-08	17	3	7	1
Cthe_0675		stage II sporulation protein M	-2.19	2.6E-05	46	16	40	14
Cthe_0691		sporulation protein YtfJ	-1.93	1.3E-08	69	29	67	28
Cthe_0813		stage IV sporulation protein B	-1.24	3.5E-07	67	45	24	16
Cthe_0838		hypothetical protein	-2.31	4.1E-08	239	75	177	56
Cthe_0839		stage III sporulation protein AG	-2.98	4.1E-10	53	10	41	8
Cthe_0840		stage III sporulation protein AF	-2.48	1.2E-07	97	27	74	21
Cthe_0841		stage III sporulation protein AE	-2.94	1.1E-09	110	22	43	9
Cthe_0842		stage III sporulation protein AD	-2.98	3.7E-06	26	5	32	6
Cthe_0843		stage III sporulation protein AC	-3.44	3.3E-08	25	3	61	8
Cthe_0844		stage III sporulation protein AB	-2.85	7.7E-06	14	3	13	3
Cthe_0845		stage III sporulation protein AA	-2.29	6.1E-07	44	14	21	7
Cthe_0929		Stage V sporulation protein S	-2.22	7.9E-29	6229	2116	11394	3985
Cthe_0960		SpoIID/LytB domain protein	0.18	1.9E-01	238	426	66	122
Cthe_0975		cell division protein FtsW	-0.97	2.0E-04	145	115	59	49
Cthe_1013		sporulation integral membrane protein YtvI	2.04	8.3E-16	20	134	9	60
Cthe_1021		stage IV sporulation protein A	-2.28	1.5E-04	692	216	226	71
Cthe_1072		sporulation protein YqfD	0.19	2.8E-01	413	752	165	308
Cthe_1073		sporulation protein YqfC	-2.34	1.7E-04	33	10	50	15
Cthe_1083		spore coat protein, CotS family	-0.80	1.7E-01	9	8	4	4
Cthe_1084		spore coat protein, CotS family	-0.88	2.6E-01	4	4	2	2
Cthe_1089		Stage V sporulation protein S	-3.02	5.2E-92	1137	219	2020	404
Cthe_1179		small acid-soluble spore protein, H-type	-0.82	4.5E-01	3	2	6	5
Cthe_1203		SpoVR family protein	-6.24	1.6E-104	7849	157	2668	55
Cthe_1229		Lipoprotein LpqB, GerMN domain	1.49	5.0E-18	76	336	58	267
Cthe_1274		sporulation integral membrane protein YlbJ	0.74	1.4E-02	43	114	15	42
Cthe_1328		stage II sporulation protein P	-2.27	5.2E-07	27	9	10	3
Cthe_1405		Spore coat protein CotH	-2.36	1.9E-20	180	54	40	13
Cthe_1759		sporulation protein YunB	-2.17	3.6E-04	11	4	7	3
Cthe_1856		small acid-soluble spore protein beta	-0.37	3.7E-01	32	40	90	114
Cthe_1920		stage II sporulation protein R	-3.11	2.6E-14	126	23	86	16
Cthe_1953		spore cortex-lytic enzyme	-1.23	1.7E-04	34	23	23	16
Cthe_2062		GerA spore germination protein	-2.38	1.3E-08	24	7	7	2
Cthe_2063		spore germination protein	-1.54	8.4E-02	4	2	2	1
Cthe_2064		germination protein, Ger(x)C family	-1.87	7.5E-03	7	3	3	1
Cthe_2071		sporulation protein YtxC	-1.62	1.7E-07	128	65	66	35
Cthe_2160		Spore coat protein CotH	0.25	1.5E-01	451	858	90	175
Cthe_2165		sporulation protein YyaC	-3.63	3.6E-16	66	8	53	7
Cthe_2166		Stage II sporulation protein E	3.03	1.1E-39	62	826	26	355
Cthe_2398		spore coat protein, CotS family	-1.18	4.2E-04	30	21	15	10
Cthe_2400		sporulation peptidase YabG	-1.84	1.3E-03	9	4	5	2
Cthe_2415		spore cortex-lytic enzyme	-1.43	6.7E-03	21	12	15	8
Cthe_2416		germination protein YpeB	-1.64	2.8E-04	46	23	16	8
Cthe_2429		sporulation integral membrane protein YtvI	-0.42	2.5E-01	20	23	9	11
Cthe_2616		stage II sporulation protein D	-1.49	3.3E-05	62	35	29	17
Cthe_2618		sporulation transcriptional regulator SpoIIID	-2.89	1.2E-04	283	54	522	99
Cthe_2628		septation protein spoVG	-0.23	3.8E-01	2053	2792	3449	4821
Cthe_2655		stage V sporulation protein T	-2.40	9.6E-04	185	51	163	45
Cthe_2659		sporulation protein YabP	-1.59	7.1E-03	22	11	37	19
Cthe_2660		spore cortex biosynthesis protein YabQ	-1.73	2.1E-03	29	14	28	13
Cthe_2681		stage II sporulation protein E	-2.82	2.0E-18	304	65	60	13
Cthe_2948		Sporulation uncharacterized protein YkwD	-1.91	1.8E-03	212	86	122	50
Cthe_3064		polysaccharide biosynthesis protein	2.25	8.8E-25	33	247	10	75
Cthe_3070		sporulation protein YyaC	-0.75	4.6E-04	157	147	125	122
Cthe_3176		small acid-soluble spore protein alpha/beta type	-1.05	1.1E-02	119	93	273	215

Locus tag	Syn.	Product (GenBank genome CP000568.1)	log2 (fold.diff.)	Signif. p	Raw gene reads PL	Raw gene reads SS	FPKM PL	FPKM SS
Cthe_3194		Sporulation lipoprotein YhcN/YlaJ-like	-1.23	1.1E-02	18	12	13	9
**Sporulation transcriptional regulation**								
Cthe_0447		RNA polymerase sigma-E factor (RNA polymerase, sigma 28 subunit, FliA/WhiG subfamily)	-1.90	9.9E-06	307	129	202	86
Cthe_0448		RNA polymerase sigma-G factor (RNA polymerase, sigma 28 subunit, FliA/WhiG subfamily)	-1.45	2.6E-03	31	18	19	11
Cthe_0812		sporulation transcription factor Spo0A	0.40	3.3E-02	124	260	73	157
Cthe_3087		sporulation transcription factor Spo0A	-0.30	2.1E-02	459	592	268	358
**Putative histidine kinases (positive and negative regulation of spo0A by phosphorylation)**								
Cthe_2695		hypothetical protein	1.95	1.9E-22	32	197	24	153
Cthe_2076		ATP-binding region ATPase domain protein	-5.35	1.4E-68	387	14	156	6
Cthe_0286		ATP-binding region ATPase domain protein	2.05	2.5E-24	36	238	13	91
Cthe_0256		ATP-binding region ATPase domain protein	-0.94	1.8E-02	22	17	5	4

Section 29.

Phage proteins

Cthe_1612		toxin secretion/phage lysis holin	-2.33	1.3E-01	1	0	1	0
Cthe_1616		phage minor structural protein	-2.22	2.2E-04	10	3	2	1
Cthe_1623		phage major tail protein, phi13 family	-1.90	2.6E-02	4	2	3	1
Cthe_1625		phage protein, HK97 gp10 family	-2.54	4.7E-03	5	1	5	1
Cthe_1626		phage head-tail adaptor	-0.24	8.6E-01	1	1	1	2
Cthe_1627		uncharacterized phage protein	-0.98	5.4E-01	1	0	1	0
Cthe_1628		phage major capsid protein, HK97 family	0.12	9.0E-01	2	4	1	1
Cthe_1630		phage portal protein, HK97 family	0.02	9.9E-01	2	3	1	1
Cthe_1643		phage-associated protein	-1.98	3.9E-35	681	270	322	133
Cthe_1645		phage-associated protein	-2.92	2.2E-03	5	1	5	1
Cthe_1649		phage antirepressor protein	-1.26	3.0E-01	2	1	1	1
Cthe_1704		toxin secretion/phage lysis holin	-2.76	1.1E-03	7	1	8	2
Cthe_1707		phage minor structural protein	-3.10	1.1E-15	37	7	9	2
Cthe_1709		phage-related protein-like protein	-0.88	6.4E-02	13	11	3	2
Cthe_1713		phage major tail protein, phi13 family	-1.04	1.3E-02	16	12	13	11
Cthe_1715		phage protein, HK97 gp10 family	-1.90	1.4E-03	10	4	12	5
Cthe_1716		phage head-tail adaptor	-1.82	9.8E-02	4	1	5	2
Cthe_1719		phage major capsid protein, HK97 family	-1.96	8.2E-05	14	5	5	2
Cthe_1721		phage portal protein, HK97 family	-2.43	2.4E-05	11	3	4	1
Cthe_1723		phage terminase, small subunit, , P27 family	-1.56	6.6E-03	9	5	9	5
Cthe_1734		phage/plasmid primase, P4 family	-3.28	1.5E-40	292	47	74	12
Cthe_1735		phage DNA polymerase	0.37	2.5E-01	30	62	8	18
Cthe_1737		phage NTP-binding protein	-0.91	3.4E-02	17	14	5	4
Cthe_1739		phage helicase	-1.34	1.8E-02	8	5	3	2
Cthe_1986		phage/plasmid primase, P4 family	-0.60	5.2E-01	3	3	1	1
Cthe_2469		phage transcriptional regulator, RinA family	-1.59	6.7E-04	14	7	15	8
Cthe_2473		Terminase small subunit	-1.06	1.4E-09	202	152	213	166
Cthe_2474		phage terminase, large subunit, PBSX family	-1.59	4.2E-18	868	448	325	175
Cthe_2475		phage portal protein, SPP1 family	-3.11	4.4E-30	278	49	91	17
Cthe_2476		phage head morphogenesis protein, SPP1 gp7 family	-2.09	2.0E-36	255	95	86	33
Cthe_2478		minor structural GP20 protein	-2.19	2.8E-11	62	21	52	18
Cthe_2479		Lj928 prophage protein	-1.87	1.5E-15	91	39	47	21
Cthe_2486		XkdM protein, phage-like element PBSX	-2.31	2.1E-07	20	6	19	6
Cthe_2488		phage tape measure protein	-2.48	1.6E-29	315	87	81	24
Cthe_2847		phage/plasmid primase, P4 family	-2.68	3.1E-04	7	2	2	0
Cthe_2865		phage/plasmid primase, P4 family	-2.70	4.3E-03	4	1	1	0
Cthe_3108		phage SPO1 DNA polymerase-related protein	-0.86	2.9E-04	208	178	172	154
Cthe_3402		phage uncharacterized protein, XkdX family	-2.31	5.2E-02	3	1	8	2
Cthe_3454		phage protein	-2.36	1.2E-01	1	0	2	0
**CRISPR-associated proteins**								
Cthe_2050		CRISPR-associated RAMP protein, SSO1426 family	-0.28	2.2E-01	99	129	56	75
Cthe_2051		CRISPR-associated RAMP protein, SSO1426 family	0.10	6.7E-01	75	127	45	79
Cthe_2057		CRISPR-associated protein, TM1812 family	0.73	5.1E-03	114	298	29	80
Cthe_2296		CRISPR-associated protein Cas2	-1.10	5.4E-03	29	21	53	40
Cthe_2297		CRISPR-associated protein Cas1	-0.84	3.3E-02	19	17	9	8
Cthe_2298		CRISPR-associated protein Cas4	0.32	6.2E-01	5	9	4	9
Cthe_2299		CRISPR-associated helicase Cas3	-0.38	2.0E-01	30	37	6	8
Cthe_2300		CRISPR-associated protein Cas5	0.46	6.5E-02	43	93	28	63
Cthe_2301		CRISPR-associated regulatory protein, DevR family	0.37	2.0E-01	35	73	19	40

Locus tag	Syn.	Product (GenBank genome CP000568.1)	log2 (fold.diff.)	Signif. p	Raw gene reads PL	Raw gene reads SS	FPKM PL	FPKM SS
Cthe_2303		CRISPR-associated protein Cas6	1.33	1.5E-06	49	196	32	132
Cthe_3201		CRISPR-associated protein Csh1 domain protein	0.40	1.5E-01	1457	3054	374	812
Cthe_3202		CRISPR-associated protein, Csh2 family	0.09	7.3E-01	676	1149	350	613
Cthe_3203		CRISPR-associated protein Cas5, Hmari subtype	0.56	1.7E-02	269	635	179	435
Cthe_3204		CRISPR-associated HD domain protein	1.01	2.9E-06	324	1039	64	212
Cthe_3215		CRISPR-associated protein, TM1812 family	-3.07	4.7E-45	218	40	82	16
Cthe_3217		CRISPR-associated protein, TIGR02710 family	-0.73	4.7E-02	21	20	8	8
Cthe_3218		CRISPR-associated protein Cas1	0.00	1.0E+00	22	34	10	17
Cthe_3219		CRISPR-associated protein Cas2	1.88	1.6E-01	0	3	0	4
Cthe_3220		CRISPR-associated protein Cas4	-1.03	1.1E-03	35	27	27	21
